# Mitochondrial Involvement in Vertebrate Speciation? The Case of Mito-nuclear Genetic Divergence in Chameleons

**DOI:** 10.1093/gbe/evv226

**Published:** 2015-11-19

**Authors:** Dan Bar-Yaacov, Zena Hadjivasiliou, Liron Levin, Gilad Barshad, Raz Zarivach, Amos Bouskila, Dan Mishmar

**Affiliations:** ^1^Department of Life Sciences, Ben-Gurion University of the Negev, Be’er Sheva, Israel; ^2^Centre for Mathematics, Physics and Engineering in the Life Sciences and Experimental Biology, UCL, London, United Kingdom; ^3^Department of Genetics, Evolution and Environment, UCL, London, United Kingdom

**Keywords:** mitochondrial DNA, mito-nuclear coevolution, nuclear DNA, RNA-seq, speciation, transcriptome

## Abstract

Compatibility between the nuclear (nDNA) and mitochondrial (mtDNA) genomes is important for organismal health. However, its significance for major evolutionary processes such as speciation is unclear, especially in vertebrates. We previously identified a sharp mtDNA-specific sequence divergence between morphologically indistinguishable chameleon populations (*Chamaeleo chamaeleon recticrista*) across an ancient Israeli marine barrier (Jezreel Valley). Because mtDNA introgression and gender-based dispersal were ruled out, we hypothesized that mtDNA spatial division was maintained by mito-nuclear functional compensation. Here, we studied RNA-seq generated from each of ten chameleons representing the north and south populations and identified candidate nonsynonymous substitutions (NSSs) matching the mtDNA spatial distribution. The most prominent NSS occurred in 14 nDNA-encoded mitochondrial proteins. Increased chameleon sample size (*N* = 70) confirmed the geographic differentiation in *POLRMT*, *NDUFA5*, *ACO1*, *LYRM4*, *MARS2*, and *ACAD9.* Structural and functionality evaluation of these NSSs revealed high functionality. Mathematical modeling suggested that this mito-nuclear spatial divergence is consistent with hybrid breakdown. We conclude that our presented evidence and mathematical model underline mito-nuclear interactions as a likely role player in incipient speciation in vertebrates.

## Introduction

The mitochondria, ancient endosymbionts which are central for cellular energy production in all eukaryotes, are operated by genes encoded by two genomes—one of bacterial origin (the mitochondrial genome, mitochondrial DNA [mtDNA]) and the nuclear genome. Vertebrate mtDNA (∼16.5 kb in humans) harbors 13 protein-coding subunits of the oxidative phosphorylation system (OXPHOS) which are essential for ATP production, and 24 RNA members of the mitochondrial translation machinery (22 tRNAs and 2 rRNAs). These genes are not sufficient to operate the mitochondrion, because most genetic information required for mitochondrial activities originates from ∼1,500 nuclear DNA (nDNA)-encoded proteins, which are translated in the cytoplasm and, in turn, are imported into the mitochondria (Pagliarini et al. 2008; Bar-Yaacov, Blumberg, et al. 2012). This requires active cross-talk between components encoded by the mtDNA and the nDNA. However, the mtDNA evolves an order of magnitude faster than the nDNA (Brown et al. 1979; Lynch et al. 2006). Therefore, genes encoding closely interacting subunits of the respiratory chain from the mitochondrial and nuclear genomes are selectively forced to retain functional mutations (Grossman et al. 2004; Gershoni et al. 2010, 2014). Hence, tight coevolution between interacting factors encoded by the nDNA and the mtDNA is essential to maintain mitochondrial activities within populations, but also at the interspecific levels (Gershoni et al. 2009; Levin et al. 2014). Accordingly, it has been shown that interfering with such coadapted mito-nuclear interactions may lead to disease (Johnson et al. 2001; Hudson et al. 2005; Rai et al. 2007; Feder et al. 2009; Potluri et al. 2009; Reinhardt et al. 2013; Strauss et al. 2013; Betancourt et al. 2014; Dowling 2014; Gershoni et al. 2014). Disrupting mito-nuclear coevolution can also lead to intrapopulation hybrid breakdown, that is, lower fitness among hybrids as reflected by alteration of fecundity, well-being, respiration, and so on (Rand et al. 2001; Ellison and Burton 2006, 2010; Barreto and Burton 2013; Burton et al. 2013; Meiklejohn et al. 2013; Zhu et al. 2014). Finally, we previously suggested that it may even lead to the emergence of new species (Gershoni et al. 2009). Recently, Trier et al. (2014) showed that mito-nuclear genotypes correlated with spatial separation between closely related sparrow species (*Passer domesticus*, *Passer hispaniolensis*, and *Passer italiae*). However, the connection between intrapopulation hybrid breakdown and mito-nuclear interactions, which may lead to speciation events, has been shown mostly in invertebrates such as *Drosophila *(Zhu et al. 2014) and the copepod *Tigriopus californicus* (Ellison and Burton 2006, 2010). Therefore, the involvement of mito-nuclear interactions in the first steps of population divergence, which set the basis to subsequent speciation events in invertebrates, is yet to be assessed.

Recently, while investigating the population genetic structure of the two Mediterranean chameleon subspecies in Israel (*Chamaeleo chamaeleon recticrista* and *Chamaeleo chamaeleon musae*), we identified a sharp mtDNA sequence divergence between two populations of morphologically indistinguishable *C.c. recticrista* across the Jezreel Valley in northern Israel (north–south of the Valley) (Bar-Yaacov, Arbel-Thau, et al. 2012). This sharp population divergence was in stark contrast to apparent mtDNA introgression observed between the *C.c. recticrista* and *C.c. musae* subspecies. The Jezreel valley was once part of an ancient marine barrier which persisted for approximately a million years and ceased to exist at least 1 million years ago (García-Veigas et al. 2009), thus allocating sufficient time for such population divergence to have occurred. We found that the two *C.c. recticrista* populations diverged by more than 330 fixed mtDNA mutations (∼2% of the entire mitochondrial genome), which resemble the amount of mtDNA changes differentiating known chameleon sister species (Bar-Yaacov, Arbel-Thau, et al. 2012). Although the north–south mtDNA differentiation was absolute without exceptions, a study of more than 300 random nDNA loci (AFLP, amplified fragment length polymorphism) did not correlate with the mtDNA divergence in chameleons, suggesting some nDNA gene flow. Notably, our previous analysis indicated that the mtDNA divergence could not be explained by mtDNA introgression, nor did it correlate with possible gender-biased dispersal differences, which are common phenomena in many instances of distribution discordance between mtDNA and nDNA markers (Bar-Yaacov, Arbel-Thau, et al. 2012; Toews and Brelsford 2012). Therefore, we asked what might have interfered with the mtDNA gene flow between the north and south populations. Because these fixed changes were distributed throughout the chameleon mtDNA sequence, with some occurring at functionally important positions, we proposed that the long-term survival of these mutations was enabled by either selective advantage or functional compensation, both within the mtDNA and between the mtDNA and the nDNA, as previously suggested (Kern and Kondrashov 2004; de Magalhaes 2005; Azevedo et al. 2006, 2009).

Recently, we de novo assembled and analyzed the *C.c. recticrista* transcriptome, thus generating the first reference for the entire transcribed portion of the chameleon genome (Bar-Yaacov, Arbel-Thau, et al. 2013). In this study, we studied RNA-seq from ten chameleons representing *C.c.recticrista* populations south and north to the Jezreel Valley. This analysis revealed candidate NSSs matching the mtDNA spatial distribution, of which the most prominent, and confirmed in larger sample size, occurred in six nDNA-encoded mitochondrial proteins. Furthermore, structural and functionality evaluation of these NSSs revealed high functional potential. Finally, mathematical modeling suggested that this mito-nuclear spatial divergence was consistent with hybrid breakdown. The implications of our results for the role of mito-nuclear interactions in incipient speciation are discussed. We especially consider the Dobzhansky–Muller model suggesting that interfering with epistatic interaction (even a single interaction) could lead to speciation events (Dobzhansky 1936; Muller 1942; Gavrilets 2003).

## Materials and Methods

### Samples Collection, RNA and DNA Extraction

**Fig. 1.—:**
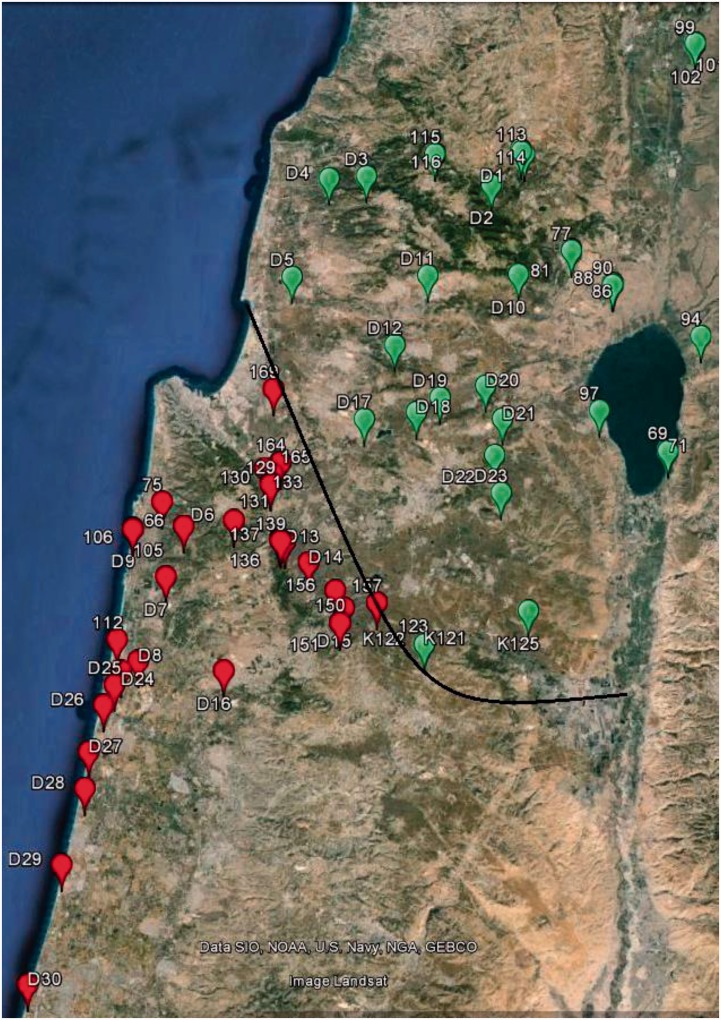
Map of sampling sites. Sampling sites marked in red and green represent chameleons with the south and north mtDNA types, respectively. Exact coordinates are specified in supplementary table S1 and file S1, Supplementary Material online. RNA-seq was performed on the following samples: D1, D2, D4, D6, D7, D8, D10, D12, D13, and D16. Black line indicates the geographic divergence boundary between chameleons with north and south mtDNA types. The figure was generated using Google earth program.

Chameleons were collected during several night expeditions at various sites throughout Israel (fig. 1, supplementary table S1 and file S1, Supplementary Material online) using a 4 × 4 vehicle equipped with strong spotlight projectors directed to shrubs and trees in which chameleons reside. Each of the captured chameleons was released at its sampling site after blood drawing (20–150 µl) from the caudal vein using 1 ml syringes. Total RNA was extracted using Perfect pure RNA kit (5 Prime, #0453274). RNA concentration was estimated using nano-drop (NanoDrop Technologies). Clear rRNA bands were visualized on a 1% agarose gel to further assure RNA sample quality. RNA samples were stored at −80 °C until usage. Blood for DNA extraction was placed in a 1.5 ml Eppendorf tube along with Gentra cell lysis solution according to manufacturer’s instructions. DNA was purified using the Puregene DNA extraction kit (Gentra, cat. # D-5500) following manufacturer’s instructions. DNA samples were stored at −20 °C until usage. All chameleon samples were collected using permits from the Israel Nature and Parks authority, numbers 2011/38142, 2012/38529, and 2013/40003.

### Massive Parallel Sequencing

Libraries for RNA-seq were prepared from chameleon RNA extracts using TruSeq RNA Paired End Kit (Illumina) according to the manufacturer protocol. RNA samples of ten chameleons were sequenced using Hi-Seq 2000 platform (Illumina, Genomics Center, Technion, Haifa). All ten libraries produced 101-bp paired-end reads. Quality scores per nucleotide position, per sample are available in supplementary table S2, Supplementary Material online.

### Transcriptome Assembly and Annotation

Each RNA-seq data set (transcriptome) was de novo assembled by CLC-Bio genomic workbench 6.01 using default parameters. Summary statistics for each assembled transcriptome are summarized in supplementary table S3, Supplementary Material online. All contigs generated from each transcriptomes were BLAST screened against the entire human Ref-seq protein database (Altschul et al. 1997). We assembled all BLAST results, sorted according to the highest BLAST score, and removed duplications in order to construct a nonredundant chameleon mRNA sequence compendium (supplementary table S4, Supplementary Material online). The mean and median lengths of the transcripts in the compendium were 2,089 and 1,590, respectively. The mean and median fraction percent length of the chameleon transcripts was 67% and 76%, respectively, as compared with the length of their human orthologs. The highest *e*-value was 10^−^^10^ (90% of the aligned sequences presented with *e*-value smaller than 7 × 10^−^^19^). The mean and median percent of identity was 71.7% and 73.7%, respectively.

### Assembly and Analysis of the Entire mtDNA Sequence from RNA-seq Data

We assembled the mtDNA sequence from the ten chameleon RNA-seq data sets. We used BWA (Li and Durbin 2009) to map the reads against our published reference sequences (GenBank accession JF317641 for “south” and JF317642 for “north” chameleons, respectively). We then converted the SAM file into a sorted BAM file using SamTools (Li et al. 2009). Finally, we used MitoBamAnnotator (Zhidkov et al. 2011) to extract the entire mtDNA sequence consensus from each sample. MEGA 6.0 (Tamura et al. 2013) was used to align reconstructed mtDNA genomes along with previously published seven whole mtDNA chameleon sequences with the following GenBank accession numbers: JF317639, sample 105; JF317640, sample 108; JF317641, sample 169; JF317642, sample 100; JF317643, sample 69; JF317644, sample 114; and JF317645, sample 123. Four publicly available *C. chamaeleon*, mtDNA sequences were also added to the alignment and tree construction: Portugal EF222198, Cyprus EF222200, Turkey 1 EF222201, and Turkey 2 EF222202. The sequence alignment encompassed >97% of the chameleon mtDNA (∼17,000 bp). Similar tree topologies were obtained using two different types of phylogenetic methods: 1) Neighbor joining, bootstrap (1,000 replicates), gap/missing data—pairwise deletion, model—maximum composite likelihood, substitution to include—all, pattern among lineages—homogeneous, rate among sites—gamma distribution; 2) maximum likelihood (ML), bootstrap (1,000 replicates), gap/missing data—partial deletion, model—Tamura–Nei model, substitution type—nucleotide, rate among sites—gamma distribution with invariant sites, ML heuristic method—Nearest-Neighbor-Interchange (NNI), automatic initial tree for ML.

### Identification of Single Nucleotide Variants Co-occurring with mtDNA Types

RNA-seq reads of each sample were compared and mapped against the chameleon mRNA sequence compendium. Single nucleotide variants (SNVs) were identified using the Probabilistic Variant Detection tool (default parameters) embedded within CLC-Bio genomic workbench 6.01. We screened the ten chameleon transcriptomes for SNVs whose geographic sample distribution correlated with the mtDNA geographic pattern. Candidate differentiating SNVs were defined as present in at least four samples harboring one type of mtDNA (e.g., northern mtDNA), and are not present in more than one sample harboring the opposite mtDNA type (e.g., southern mtDNA).

### Gene Ontology Analysis

We subjected all identified 190 genes to Gene Ontology Consortium (GOC) pipeline using the PANTHER classification system (Ashburner et al. 2000; Thomas et al. 2003). Specifically, we extracted the GenBank accession numbers (GIs) of all genes and used them for GOC enrichment analysis (http://geneontology.org/, last accessed December 1, 2015). By employing PANTHER we chose three lines of analysis: Biological processes, molecular function, and cellular components. For each of the analyses a list of categories with significant enrichment was reported, while employing Bonferroni correction for multiple testing. The complete list of enriched categories is available in supplementary tables S5–S8, Supplementary Material online.

### Sanger Sequencing of 14 nDNA-Encoded Mitochondrial Genes

We used our in-house constructed bioinformatics tool, LEMONS (Levin et al. 2015, http://lifeserv.bgu.ac.il/wb/dmishmar/pages/lemons.php, last accessed December 1, 2015), to identify splice junctions in 14 nDNA-encoded mitochondrial genes. In brief, the tool is based on the logic that exon–intron architecture is conserved during vertebrates evolution (Rogozin et al. 2003; Carmel et al. 2007). LEMONS utilize the known exon–intron architecture in several vertebrate model species to create a BLAST database with splice junction information in combination with splice junctions’ motif search. All the mRNA sequences were then compared with the human Ref-seq database as well as to five additional genomic databases (from *Mus musculus*, *Gallus gallus*, *Anolis carolinensis*, *Xenopus tropicalis*, and *Danio rerio*) to identify splice junctions. The executable file and a user manual for LEMONS are available for download at http://lifeserv.bgu.ac.il/wb/dmishmar/pages/lemons-temp.php (last accessed December 1, 2015). Splice junction identification enabled us to design polymerase chain reaction (PCR) primers for amplification and subsequent sequencing of DNA fragments encompassing the identified nonsynonymous SNVs (supplementary table S9, Supplementary Material online, lists the sequences of all primers used for PCR amplification and sequencing). PCR reactions for all tested genes were conducted in a 20 µl reaction mix containing 5 pmol of forward and reverse primers (each), 1.25 U of Taq polymerase (Bio-Lab), 1 × reaction buffer (Bio-Lab), 2 mM MgCl_2,_ and 0.2 mM dNTP mix, as well as ∼50 ng DNA as template. Alternative amplification was performed using Phusion polymerase (Thermo) in a 20 µl reaction mix containing 5 pmol of forward and reverse primers each, 1.25 U of Phusion polymerase (Thermo), 1 × reaction buffer (Thermo), 0.2 mM dNTP mix, and ∼50 ng DNA as template. PCR conditions and primers used are provided in supplementary tables S9 and S10, Supplementary Material online. Table 1 lists the accession numbers of human proteins used to define the nucleotide positions analyzed in each of the studied genes.

**Table 1:** NSS in nDNA-Encoded Mitochondrial Proteins with Correlated Genotype Distribution to the mtDNA Geographic Pattern^a^

Gene Name	Sample Size	Number of NSS	*P* value	Bonferroni *P* value	Fst	Human Protein Ref-seq Accession Number
***POLRMT***	70	2	0.000010	0.00014	0.18	NP_005026.3
***ACO1***	69	1	0.000025	0.00035	0.29	NP_002188.1
***NDUFA5***	70	1	0.000044	0.00062	0.25	NP_004991.1
***LYRM4***	70	1	0.001700	0.02380	0.14	NP_065141.3
***MARS2***	70	3	0.002160	0.03024	0.14	NP_612404.1
***ACAD9***	61	1	0.003300	0.04620	0.11	NP_054768.2
*AARS2*	69	1	0.006300	>0.05	—	NP_065796.1
*ETFA*	70	1	0.008500	>0.05	—	NP_000117.1
*MRPL30*	70	1	0.014000	>0.05	—	NP_660213.1
*P32*	70	1	>0.05	>0.05	—	NP_001203.1
*TCIRG1*	70	1	>0.05	>0.05	—	NP_006010.2
*SDHC*	53	1	>0.05	>0.05	—	NP_002992.1
*TAP1*	Not included in the final analysis				—	NP_000584.2
*PHYH*	Not included in the final analysis				—	NP_006205.1

^a^Sample size, total number of genotyped chameleons; number of NSS, number of NSS per gene; *P* value, chi-square *P* value; Bonferroni *P* value, *P* value after Bonferroni correction; Fst, calculated Fst value for each gene using Genepop (Rousset 2008).

Notably, due to technical reasons we were unable to analyze the genes *PHYH* and *TAP1.* Specifically, we were unable to amplify *PHYH* with several alternative primer pairs. Additionally, after increasing the sample size for *TAP1*, and sequencing individual samples that were included in the analysis of the transcriptomes, we were unable to corroborate the presence of the *TAP1* genotype identified in the transcriptomes. Therefore we excluded *PHYH* and *TAP1* from further analyses.

All PCR conditions, amplification, and sequencing primers for all genes are indicated in supplementary table S10, Supplementary Material online. In several cases after PCR amplifications and initial sequencing reactions, we sought to improve the quality of sequencing reads. For these cases we designed additional primers for amplification or sequencing.

PCR products of all tested genes were visualized on an EtBr-stained 1% agarose gel, purified using Wizard SV Gel and PCR Clean-up system (Promega #A9282), following the manufacturer instructions, and sequenced (ABI 3100) using the above-mentioned specific primers (BGU sequencing facility).

Notably, many of the sequences generated were shorter than 200 bp, because we were interested in the analysis of specific mutations. Such short sequenced fragments did not match GenBank criteria for submission and therefore were not deposited in GenBank. Nevertheless, sequences are available and will be shared upon request.

### Restriction Fragment Length Polymorphism Analysis

Restriction fragment length polymorphism (RFLP) was conducted after sequencing of 20 (*N* = 20) specimens in all genes (except for ETFA where fewer sequences were available) to identify the alleles in the tested samples. RFLP was performed in the following genes to determine allele compositions (see supplementary table S11, Supplementary Material online, for restriction mix and conditions): POLRMT (mutation at position 1218)—both *Taq*^α^I (New England BioLabs #R0149S) and *Mluc*I (New England Biolabs #R0538S); MRPL30—*Pst*I (New England BioLabs #R0140S); ETFA—*Hind*III (Thermo #ER0501); and LYRM4—*Apek*I (New England BioLabs #R0643S).

### Assessing Possible Linkage between the Six nDNA Genes Which Correlated to the mtDNA Pattern

It is possible that association between genotype distributions of the analyzed genes was skewed if these genes are linked. Because the chameleon karyotype is yet to be resolved we asked whether the analyzed genes reside on syntenic chromosomal fragments. To this end, all six nDNA genes showing association with the mtDNA pattern (*POLRMT*, *NDUFA5*, *ACO1*, *LYRM4*, *MARS2*, and *ACAD9*) were examined in the NCBI protein data base for chromosomal locations. First, we determined the loci of the genes in the *A. carolinensis* genome, and were able to retrieve information only for *NDUFA5* and *ACAD9.* We therefore examined the chromosomal mapping of all six genes in humans, chicken, and zebra finch, being the closest species to chameleons having sufficient chromosomal mapping information.

### Fst Calculation and Statistical Analysis

We used DNASP 5.0 to assess the mutation patterns and Fst between the tested populations (Librado and Rozas 2009). Fst scores range from 0 to 1, that is, from complete allele sharing to complete population divergence with each population having their own fixed alleles. Qualitative guidelines for the interpretation of Fst are as follows: 0–0.05 minute genetic differentiation; 0.05–0.15 moderate genetic differentiation; 0.15–0.25 high genetic differentiation; 0.25 profound genetic differentiation (Wright 1978). We used chi-square test for independence to determine whether an NSS distribution correlates to the mtDNA pattern (table 1). Accordingly, we compared the null hypothesis (i.e., no population differences) with the possibility that the two populations diverge. Specifically, the null hypothesis states that the distribution of NSS should match random differentiation of two groups originating from the same population. *P*-values were calculated using STATISTICA v.11. Additionally, Fst values for each differentiating nDNA NSS were calculated using Genepop (Rousset 2008; table 1).

### Assessing the Functionality of NSS

Three basic measurements were employed to assess the functionality of the identified NSSs in all 13 mtDNA protein-coding genes and 6 nDNA-encoded mitochondrial protein-coding genes. We calculated the conservation index (Glaser et al. 2003), the Sorting Intolerant from Tolerant (SIFT) pathogenicity score of amino acid substitutions (http://sift.bii.a-star.edu.sg/, last accessed December 1, 2015) (Kumar et al. 2009), and PANTHER score. For the conservation index we generated sequence alignment files using MAFFT (mafft.cbrc.jp/alignment/server/) and MEGA 6 (Tamura et al. 2013) to visualize and analyze the alignment, with minor manual corrections, using orthologous sequences from ∼120 lizard species (Order Squamata) for the mtDNA-encoded proteins (supplementary table S12, Supplementary Material online) and 78-106 vertebrate species for each of the nDNA-encoded proteins (table 3). The conservation index was calculated by ConSurf (consurf.tau.ac.il, last accessed December 2, 2015) (Glaser et al. 2003) using default settings. Conservation index scores *X* ≥ 7 were considered as having the highest functionality, being within 1 SD from the mean conservation index scores of mtDNA disease-causing mutation (8.4 ± 1.8, according to Levin et al. 2013). As previously suggested (Kumar et al. 2009), SIFT pathogenicity scores ≤0.05 were considered as having the highest functionality. The third functionality test was Panther *P*-deleterious score (Thomas et al. 2003), while using the default cutoff value of 0.5.

### Structural Modeling and Examination

The three-dimensional (3D) protein structures of POLRMT, MARS2, ACO1, and ACAD9 were modeled using Swiss-Model (Schwede et al. 2003). NDUFA5 and LYRM4 did not have any available or closely related structures to enable modeling. For POLRMT the human structure was used to predict the chameleon structure and was overlapped with T7 RNA polymerase (PDB—4BOC and 1CEZ; Cheetham et al. 1999; Ringel et al. 2011; Schwinghammer et al. 2013). The *Leishmania major* MARS structure was used to predict the chameleon structure and was overlapped with *Aquifex aeolicus* MARS Met-tRNA (PDB—3KFL and 2CT8; Nakanishi et al. 2005; Larson et al. 2011). The human ACO1 structure was used to predict the chameleon structure (PDB—2B3Y; Dupuy et al. 2006). For ACAD9 the human structure of ACADVL was used to predict the chameleon structure (PDB—2UXW, unpublished). All models were visualized and examined using WinCoot (Emsley and Cowtan 2004) and figures were prepared by PyMOL (DeLano 2002).

### Correlated Mutations Test

We employed the correlated mutation test (webclu.bio.wzw.tum.de:18080/ComplexCorr/instruction.html, last accessed December 2, 2015) as described previously (Gershoni et al. 2010). In brief, we calculated correlated mutation scores using two methods: observed minus expected squared (OMES) and explicit likelihood of subset covariation (ELSC). Each score above 5 and 20 in the OMES and ELSC tests, respectively, implied coevolution between the tested proteins and 88% chances for protein–protein interactions. We calculated the top scores of the predicted correlation between NDUFA5 and each of the seven mtDNA-encoded complex I subunits (ND1–ND6, ND4L).

### Mathematical Model

We consider two populations, *P*_S_ and *P*_N_, harboring different mtDNAs denoted by *m*_S_ and *m*_N_, for the south and north, respectively. We assume that *n* nDNA loci interact with the mitochondria and that mito-nuclear interactions are independent of one another. We also assume that the nDNA loci are homozygous in the two populations and the optimal alleles depend on the mtDNA background so that (*a*_1_*a*_1_, *a*_2_*a*_2_,…, *a_n_a*_n_) and (*A*_1_*A*_1_, *A*_2_*A*_2_,…, *A_n_A_n_*) denote the nDNA alleles matched to the north and south mtDNA, respectively. We define the fitness of locus *i* against each mitochondrial background by *w*(*a_i_a*_i_, *m_N_*) = *w*(*A_i_A*_i_, *m_S_*) = 1, *w*(*A_i_a_i_*, *m_S_*) = *w*(*A_i_a_i_*, *m_N_*) = 1 − *s*_1_, and *w*(*A_i_A_i_*, *m_N_*) = *w*(*a_i_a_i_*, *m_S_*) = 1 − *s*_2_. Assuming that mito-nuclear interactions are independent of one another, organism fitness is equal to $\prod_{i=1}^{n}w_{i}$, where *w_i_* is the fitness with respect to the *i*th interaction. Using this formulation, we can compute the expected fitness of F1 and F2 individuals, and of F1 individuals backcrossed to the parental populations as follows.

The fitness of F1 individuals is fixed, as all nDNA loci are heterozygous, and is given by

(1) $$W_{1}={\left(1-S_{1}\right)}^{n}$$

Note that *s*_1_ can be very small or negligible because a single coadapted nDNA gene is always present at each locus; the value of *s*_1_ depends on the dominance dynamics between the two alleles. The expected fitness for F2 individuals is given by

(2) W2=∑j=0n−i∑i=0nP(x=i,y=j,z=n−i−j)(1−s2)i(1−s1)j=∑j=0n−i∑i=0n(n!i!j!(n−i−j)!)(14)i(12)j(14)n−i−j(1−s2)i(1−s1)j=∑j=0n−i∑i=0n(n!i!j!(n−i−j)!)(12)2n−j(1−s2)i(1−s1)j

where *x* is the number of homozygotes mismatched to the mtDNA of the zygote, *y* is the number of heterozygotes, and *z* is the number of homozygotes that match the mitochondrial background. The expected fitness of F1 males backcrossed to either population and F1 females backcrossed to their maternal population is given by

(3) W3=∑i=0nP(x=i,y=n−j)(1−s1)i=∑i=0n(in)(12)n(1−s1)i

where *x* is the number of heterozygous loci and *y* is the number of homozygotes matching the mitochondrial background. Note that no mismatched homozygotes are now possible and fitness can be restored, especially if the heterozygous disadvantage is small. Finally, the expected fitness of females backcrossed to their paternal population is given by

(4) W4=∑i=0nP(x=i,y=n−i)(1−s1)i(1−s2)n−i=∑i=0n(in)(12)n(1−s1)i(1−s2)n−i

where *x* is the number of heterozygous loci and *y* is the number of homozygotes not matching the mitochondrial background. No fully matched homozygotes are now possible and zygote fitness is expected to decline steeply as the number of nDNA loci interacting with the mitochondria increases. If disturbance of the interaction is crucial (large *s*_2_), even a single interacting locus can cause significant reproductive isolation for backcrossed females. Our formulation is directly applicable to loci favoring different homozygotes depending on the mitochondrial background. The alleles for POLRMT 1090 found in this study are suggestive of such a pattern. We also extended this to consider polymorphic nDNA loci. This may lead to a less prominent reduction in fitness of hybrids and backcrosses (see supplementary data for further details).

## Results

### Chameleon Reference Transcriptome

**Fig. 2.—:**
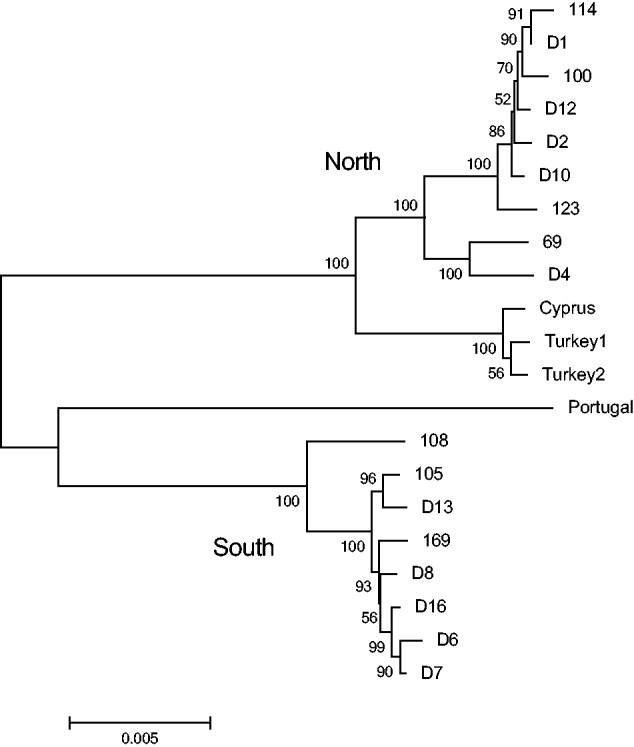
Maximum likelihood tree based on 21 whole mtDNA sequences. Bootstrap scores of 1,000 replicates are indicated near the branches. Sequences from samples marked with a “D” were generated in this study, while the rest are from our previous work (Bar-Yaacov, Arbel-Thau, et al. 2012). GenBank accession numbers are the following: Portugal EF222198, Cyprus EF222200, Turkey 1 EF222201, and Turkey 2 EF222202. The tree is drawn to scale, with branch lengths calculated using the average pathway method. Fst between the two populations based on the whole mtDNA sequences was 0.885. For accession numbers, see Materials and Methods section. For sample collection sites, see figure 1 and supplementary table S1, Supplementary Material online.

The mtDNA divergence between the *C.c. recticrista* populations across the Jezreel Valley included multiple mutations in sites with potential functionality (Bar-Yaacov, Arbel-Thau, et al. 2012). We asked whether this sharp north–south divergence has been maintained, at least in part, due to functional compensation via nDNA gene loci. We initially set to identify nDNA-encoded SNVs exhibiting geographic distribution that correlates with the divergence pattern of chameleon mtDNAs. To this end, we sequenced and de novo assembled ten chameleon transcriptomes (supplementary tables 1 and 2, Supplementary Material online) from diverse collection sites in northern Israel (fig. 1). Next, we used the RNA-seq reads to extract and assemble more than 97% (∼17,000 bp) of the mtDNA sequences in each of the tested samples. These sequences were aligned along with our previously published *C. c. recticrista* mtDNA sequences (Bar-Yaacov, Arbel-Thau, et al. 2012), as well as with four available non-Israeli *C. chamaeleon* sequences (Macey et al. 2008). Phylogenetic analysis validated our previous identification of two distinct mtDNA chameleon clades harboring either the north or south mtDNA types (fig. 2). Furthermore, this north–south divergence was deep especially in comparison with the non-Israeli chameleons. Additionally, the multiple sequence alignment revealed 335 fixed mtDNA SNVs differentiating the two populations, of which 63 were nonsynonymous SNVs (NSSs), with some showing high functionality (supplementary table S12, Supplementary Material online). Finally, we generated a nonredundant reference chameleon transcriptome encompassing 16,536 human orthologous transcripts, which served as a reference for further analyses and comparisons (supplementary table S4, Supplementary Material online).

### Nonsynonymous SNVs in nDNA-Encoded Mitochondrial Proteins Are Candidates for Mito-nuclear Compensation

To identify candidate SNVs in nDNA-encoded genes that differentiate the chameleons according to their mtDNA types (i.e., north and south to the Jezreel Valley), we mapped all the RNA-seq samples against the reference mRNA transcriptome. Candidate nDNA-encoded SNVs were defined as those present in at least four samples harboring a certain mtDNA type (i.e., north or south), and present in no more than one sample harboring the other mtDNA type. This approach resulted in the identification of 3,333 SNVs across the entire chameleon transcriptome, including 254 NSSs in 190 genes (supplementary table S5, Supplementary Material online).

We applied Gene Ontology (GO) analysis to all identified 190 genes. Because of data availability, our analysis focused on 178 genes which were identified by the used pipeline (supplementary table S5, Supplementary Material online). Of these nDNA-encoded genes, the top enriched biological processes were “RNA metabolic processes” and “transcription DNA template” (supplementary table S6, Supplementary Material online). Furthermore, the most prominent GO categories of molecular functional analysis were “transcription factors” and “nucleotide binding proteins”(supplementary table S7, Supplementary Material online) and the major localization signal comes from the nucleus in the cellular component analysis (supplementary table S8, Supplementary Material online). Therefore, although our previous analysis underlined the putative functional involvement of the mitochondria in the population divergence in Israeli chameleons, we do not exclude the possibility that additional specific genes with nonmitochondrial function contributed to the observed population differentiation.

Next, we aimed toward prioritizing the best candidates to functionally compensate for the observed mtDNA divergence pattern in chameleons. Accordingly, while inspecting the list of 190 genes we found that 14 genes had clear orthologs in the MitoCarta database (Pagliarini et al. 2008; table 1), hence encoding mitochondrial proteins. Because our main hypothesis focused on mito-nuclear interactions, we carried out further analysis only on the 14 nDNA-encoded mitochondrial transcripts.

We next aimed to validate the association between the distribution of the NSS alleles of the 14 nDNA–mitochondrial transcripts with the mtDNA north–south differentiation pattern. To this end, we genotyped these 14 genes in an enlarged sample of chameleons (∼70 DNA samples) available to us representing the north–south chameleon groups. As RNA-seq data harbor mature mRNA sequences, there was a need to map exon–exon junctions prior to primer design for subsequent PCR amplification from DNA samples. We therefore designed a bioinformatics tool, LEMONS, that considers the striking evolutionary conservation of gene architecture to identify exon–exon junctions in mRNA sequences lacking sequenced genomic reference (Levin et al. 2015, http://lifeserv.bgu.ac.il/wb/dmishmar/pages/lemons.php, last accessed December 1, 2015). Design of PCR primers using LEMONS resulted in successful PCR amplification followed by either Sanger sequencing or RFLP analysis of regions encompassing the NSSs in the entire tested gene set. Due to technical reasons we decided not to include the genes *PHYH* and *TAP1* in further analyses (see Materials and Methods). Statistical analysis (chi-square test of independence followed by Bonferroni correction for multiple testing) confirmed the association with the north–south mtDNA differentiation pattern for the genes *POLRMT*, *NDUFA5*, *ACO1*, *LYRM4*, *MARS2*, and *ACAD9* (table 1)*. *Each of these six genes localize to different chromosomes in humans, chicken, and the zebra finch, thus likely excluding intergene linkage and subsequent synteny in chameleons. Fst analysis calculated for all six genes showed moderate to high gene flow arrest between north and south genotypes (table 1).

We next set forth to examine the specific distribution and nature of each NSS within the corresponding protein sequences. For the sake of simplicity, all positions were numbered according to the orthologous human proteins (table 1). The distribution of all SNVs, their position in the transcript, and their consequent amino acid substitutions are listed in table 2. Notably, in the NSS at POLRMT position 1090, we observed the same proportion of heterozygotes in both the south and north populations, but exactly the opposite distribution in the two homozygous states, that is, 2 and 16 homozygotes for glutamine (Q) in the south and north populations, respectively, and exactly the opposite in the homozygous state of the leucine (L) allele. In POLRMT position 1218, and ACO1 position 601, not a single homozygote for asparagine (N) and histidine (H) alleles, respectively, were found in the south population, while the northern chameleon population had 4 and 7 individuals with N and H, respectively. The NSS identified in NDUFA5 position 95 revealed the opposite type of distribution: Not a single homozygote for valine (V) was observed in the north population, while five chameleons were homozygous for this amino acid in the south population. Considering the three NSSs identified in MARS2 (positions 298, 345, and 498—all belonging to an apparently single haplotype), all were in a homozygous state in 30 of the 35 northern chameleons (only one homozygote for the haplotype containing the other alleles), while the 35 southern chameleons had significantly more heterozygotes and homozygotes for the opposite haplotype (which was rare in the north) (table 2).

**Table 2:** Distribution of Each NSS in the Six analyzed nDNA-Encoded Mitochondrial Genes

Protein, Amino Acid Position, and mtDNA Type	Homozygote 1	Heterozygote	Homozygote 2	Total Samples Analyzed
POLRMT 1090	A (Q)	A/T (Q/L)	T (L)	70
North	16 (0.457)	17 (0.485)	2 (0.057)	35
South	2 (0.057)	17 (0.485)	16 (0.457)	35
POLRMT 1218	A (N)	A/G (N/D)	G (D)	70
North	4 (0.114)	21 (0.6)	10 (0.286)	35
South	0 (0)	7 (0.2)	28 (0.8)	35
ACO1 601	A (H)	A/G (H/R)	G (R)	69
North	7 (0.2)	16 (0.457)	11 (0.314)	35
South	0 (0)	5 (0.147)	30 (0.882)	34
NDUFA5 95	C (A)	C/T (A/V)	T (V)	70
North	30 (0.857)	5 (0.147)	0	35
South	12 (0.343)	18 (0.514)	5 (0.147)	35
LYRM4 51	A (A)	A/C (A/E)	C (E)	70
North	8 (0.228)	20 (0.571)	7 (0.2)	35
South	23 (0.657)	9 (257)	3 (0.857)	35
MARS2 298	C (L)	C/T (L/F)	T (F)	67
North	30 (0.857)	4 (0.114)	1 (0.028)	35
South	14 (0.437)	13 (0.406)	5 (0.156)	32
MARS2 345	G (G)	G/A (G/R)	A (R)	70
North	30 (0.857)	4 (0.114)	1 (0.028)	35
South	19 (0.543)	12 (0.343)	4 (0.114)	35
MARS2 498	G (E)	G/A (E/K)	A (K)	70
North	30 (0.857)	4 (0.114)	1 (0.028)	35
South	19 (0.543)	12 (0.343)	4 (0.114)	35
ACAD9 503	G (M)	G/A (M/I)	A (I)	61
North	29 (0.966)	1 (0.033)	0 (0)	30
South	21 (0.677)	10 (0.323)	0 (0)	31

Note.—Amino acid alleles and allele frequencies are given in in parentheses.

**Table 3:** Functionality Assessment of the NSS^a^

Protein Name	Human Position	AA North	AA South	PANTH	SIFT	CI	AA Distribution	Number of Species	Species used
POLRMT	1090	Q	L	0.26	0.51	1	V (32), **Q (21.8), L (19.2)**, M (15.4), S (6.4), T (2.6), C (1.3), I (1.3)	78	46 mammals, 16 birds, 2 reptiles, 2 amphibians, 12 fish
					Q>L				
POLRMT	1218	N	D	—	0.38	**7**	**D (69.2), N (29.5), K (1.3)**	78	46 mammals, 16 birds, 2 reptiles, 2 amphibians, 12 fish
					D>N				
ACO1	601	H	R	0.2	**0.03**	1	**R (90.6),** Q (4.7), **H (2.8)**, C (0.95), K (0.95)	106	71 mammals, 16 birds, 3 reptiles, 2 amphibians, 14 fish
					**R > H**				
NDUFA5	95	A	V	0.11	0.19	1	I (30.1), L (26.2), **V (15.5),** M (11.6), S (5.8), R (4.9), **A (3.9)**, K (1), Y (1)	103	69 mammals, 17 birds, 2 reptiles, 2 amphibians, 13 fish
					R > A				
					0.57				
					R > V				
LYRM4	51	A	E		0.21	5	**E (65.8)**, K (15.3), Q (4.7), **A (5.9)**, T (4.7), R (1.2), S (1.2), V (1.2)	85	57 mammals, 13 birds, 2 reptiles, 2 amphibians, 11 fish
					E > A				
MARS2	298	L	F	0.26	0.2	1	K (49), G (15.3), E (10.2), R (6.1), N (5.1), S (4.1), Q (3.1), **L (2.05)**, D (2.05), A (1), H (1), T (1)	98	63 mammals, 16 birds, 3 reptiles, 2 amphibians, 14 fish
					K > L				
					**0.03**				
					**K > F**				
MARS2	345	G	R	**0.57**	**0**	**9**	**G (100)**	98	63 mammals, 16 birds, 3 reptiles, 2 amphibians, 14 fish
					**G > R**				
MARS2	498	E	K	0.31	0.09	1	**E (65.3)**, **K (13.3)**, R (11.2), G (3.1), D (2.05), N (2.05), Q (1), S (1), T (1)	98	63 mammals, 16 birds, 3 reptiles, 2 amphibians, 14 fish
					E > K				
ACAD9	503	M	I	0.4	**0**	5	E (77.6), Q (20.4), N (1), L (1)	98	65 mammals, 15 birds, 3 reptiles, 2 amphibians, 13 fish
					**E > M**				
					**E > I**				

^a^Human position, position in the human protein ortholog; AA north, the prominent amino acid form in the northern population; AA south, the prominent amino acid form in the southern population; PANTH, PANTHER score; SIFT, SIFT score in the vertebrate MSA; CI, conservation index score extracted from the MSA; AA distribution, amino acid distribution in the vertebrate MSA (percentage in parenthesis). Bold in the SIFT, PANTH, and CI columns—values that passed the threshold of significance. Bold in the column entitled “AA Distribution”—amino acid variants found in chameleons but also in other species.

### North–South NSS Could Be Functionally Divergent

**Fig. 3.—:**
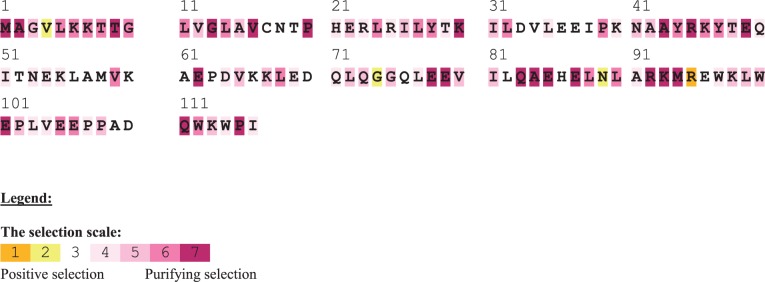
Position 95 in NDUFA5 is positively selected in vertebrates. Notably, only position 95 gained the highest score for positive selection while analyzing multiple sequence alignment of 100 vertebrates. Orange and purple correspond to the highest positively and negatively selected scores, respectively.

In order to assess the potential function of the identified NSS, we generated multiple vertebrate sequence alignment (MSA) for each of the analyzed proteins, and employed several prediction methods (SIFT, PANTHER, and conservation index; table 3). The two most evolutionarily conserved NSSs were POLRMT position 1218 and MARS2 position 345 (conservation indexes of 7 and 9, respectively), with the latter (MARS2 position 345) also exhibiting high functionality PANTHER and SIFT scores. ACO1 position 601, ACAD9 position 503, and MARS2 position 298 (for the latter—only the Phenylalanine allele) were highly scored by SIFT. Investigating the vertebrate sequence alignment of POLRMT (*N* = 78 species) revealed that position 1218 harbored either an aspartate (D) or an asparagine (N), which differ in their physical–chemical properties. These are the exact NSSs differentiating the north from the south mtDNA clusters. Sequence alignment of 98 available vertebrate MARS2 sequences revealed that position 345 mostly harbored a glycine (G) which was the most frequent amino acid in the northern chameleon group, while arginine (R) occupied this position in the southern group. Finally, SELECTON analysis (Stern et al. 2007) of 100 nonredundant vertebrate sequences revealed that NDUFA5 position 95 that differentiated the north from the south chameleons got the highest score available for positive selection (fig. 3). All other NSS variants did not cross the score threshold in any of the functionality assessment tests using the vertebrate MSA, and had either average (LYRM4 and ACAD9) or low conservation index scores (POLRMT1090, ACO1, and the other two NSSs in MARS2).

**Fig. 4.—:**
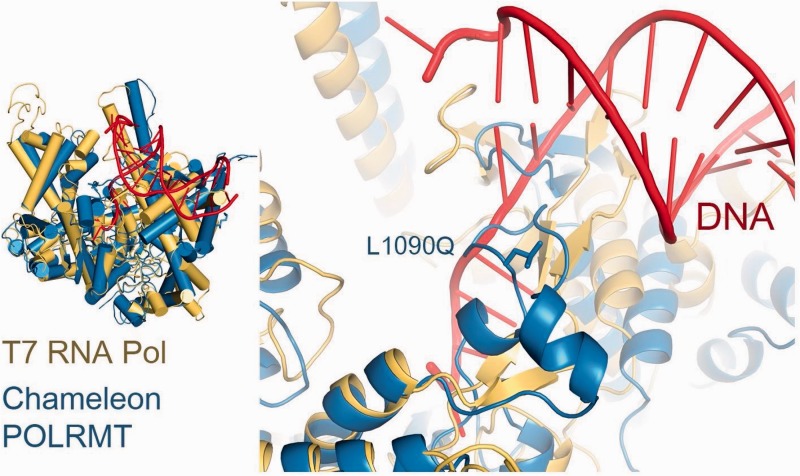
Structural modeling of POLRMT NSS in chameleons. Left, chameleon POLRMT model (blue) superimposed on the promoter(red)-bound T7 RNA polymerase (yellow, 1CEZ). The NSS in position 1090 is shown to be within the specificity loop of POLRMT and near the promoter region.

As a complementary approach to the above, we asked whether any of the identified nDNA NSS that differentiate the south and north chameleon populations potentially affect protein structure-function relationships. Although modeling these NSSs on available 3D structures (POLRMT, MARS2, ACO1, and ACAD9), we found that some of the identified NSSs may affect the proteins at the structural level: POLRMT position 1090 is located at the specificity region of the protein (Arnold et al. 2012), which is within the promoter recognition domain of this protein. Our model suggested that replacing the glutamine (prevalent in the north population) for a hydrophobic leucine (prevalent in the south population) could have affected the binding affinity or mtDNA promoter recognition by POLRMT (fig. 4). This amino acid replacement correlated with 46 fixed mutations in the mtDNA control region that differentiated the two chameleon populations, which may contain the best candidate interacting bases. Once mtDNA transcription assays are available for chameleon mitochondria, the exact location of the chameleon mtDNA promoters will be revealed, thus allowing testing for causality among control region mutations. The NSS at POLRMT position 1218, which got high functionality test scores (see above), was mapped to the protein surface, and its two alleles, namely asparagine (enriched in the north population) or an aspartic acid (enriched in the south population), did not alter the conformation of the protein, yet may potentially interfere with protein–protein interactions. The NSS at MARS2 position 345 was in close proximity to the tRNA negatively charged backbone. Replacement of the small glycine (enriched in the north population) by a large and positively charged arginine (enriched in the south population) may have increased the affinity of MARS2 to the tRNA (supplementary fig. S1, Supplementary Material online). Replacement of the negatively charged glutamate (prevalent in the north) for a positively charged lysine (prevalent in the south) in the NSS at MARS2 position 498, which is in the vicinity of the tRNA anticodon loop, likely increased the positive electrostatic distribution, to allow better binding to the tRNA (supplementary fig. S1, Supplementary Material online).

Finally, in order to deepen our understanding of the correlated response of the nucleus to the sharp mtDNA divergence, we focused on NDUFA5, which is a structural member of OXPHOS complex I. As mentioned above, NDUFA5 position 95 that divided the south and north chameleon populations was positively selected, thus implying its adaptive response to functional changes in the mtDNA. Of the mtDNA nonsynonymous changes that divided the south–north chameleon populations, three mutations had the highest functionality scores, all occurring in complex I subunits (two in ND5 and one in ND6). In order to decipher which of these two complex I subunits are best candidate to interact with NDUFA5, we used our previous experimentally supported correlated mutations test (Gershoni et al. 2010). Our analysis gave the highest OMES score (26.7) for ND5 out of the seven mtDNA-encoded proteins. Taken together, ND5 constitutes the best candidate to coevolve with NDUFA5, and hence most likely reflecting functional interaction.

### Mathematical Model Suggests that Hybrid Breakdown Explains Mito-nuclear Divergence in Chameleons

**Fig. 5.—:**
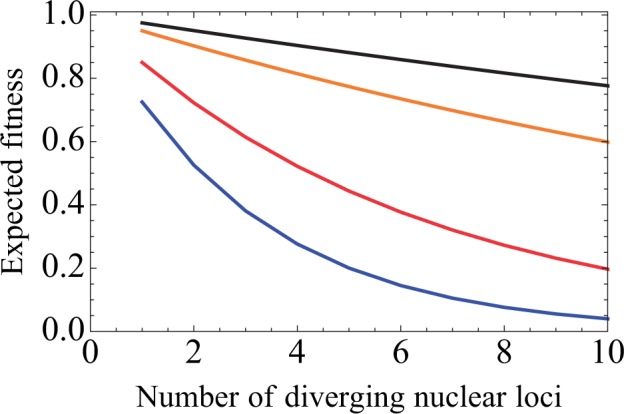
The expected fitness curves of different hybrid crosses as a function of the number of diverging nDNA loci interacting with the mtDNA. Values were computed using equations (1)–(4) (see Methods). Orange: F1 individuals; black: F1 males backcrossed to either parental population or F1 females backcrossed to their maternal population; red: F2 hybrids; blue: F1 females backcrossed to their paternal population. These curves were calculated under the assumption that the cost for heterozygotes (*s*_1_) equals to 0.05 and the cost for a homozygote against the wrong mitochondrial background equals to 0.5. Variation in these values affects the steepness of the fitness curves but their order is maintained.

To gauge whether the divergence between the north and south populations (relative to the Jezreel Valley) can be understood in light of mito-nuclear breakdown, we constructed a simple model of mito-nuclear genotype interactions. We considered two populations harboring distinct mitochondrial genomes and assumed that a number of nDNA loci interact with the mitochondria. We also assumed that these loci are homozygous in the two populations and that the optimal alleles depend on the mitochondrial background. We denote the cost suffered by heterozygotes as *s*_1_, and homozygotes against the wrong mitochondrial background as *s*_2_. We consider mito-nuclear interactions that are independent from one another. Notably, this formulation is conceptually similar to that of Orr (1995) for Dobzhansky–Muller incompatibilities, but modified to incorporate mito-nuclear interactions instead of nuclear–nuclear interactions (see Methods, supplementary methods and supplementary fig. S2, Supplementary Material online). Figure 5 shows the expected fitness for F1 hybrids, F2 hybrids, and F1 hybrids backcrossed to the parental populations, as a function of the number of diverging mito-nuclear interactions. The viability of F1 backcrosses depends heavily on the sex of the F1 hybrid: F1 females backcrossed to their paternal population will produce offspring with lower expected fitness. F1 males backcrossed to either parental population and F1 females backcrossed to their maternal population can largely restore their fitness loss. These findings suggest that F1 hybrids, suffering minor or no fitness loss, can be sources of nDNA gene flow from one population to the other. Mitochondrial genes, on the other hand, can hardly move between the two populations because female hybrids can only produce viable offspring with males from their maternal population (i.e., having nDNA genes compatible with their mitochondrial background). Naturally, the more nDNA loci that differentially interact with the mitochondria in the two populations, the more stringent the flow of nDNA genes from one population to the other and the stronger the presumed hybrid breakdown. Notably, even a single nDNA–mtDNA interaction can cause some reproductive isolation, if its interaction with the wrong mitochondria obscures vital functions (i.e., very high *s*_2_; fig. 5).

## Discussion

In this study, we asked whether selection over mito-nuclear interactions could explain the sharp mtDNA sequence divergence between chameleon populations north and south of the Jezreel Valley, which was an ancient marine barrier up until ∼1 million years before present (Bar-Yaacov, Arbel-Thau, et al. 2012). Transcriptome-wide sequence analysis representing the north and south chameleons revealed six nDNA-encoded mitochondrial transcripts harboring NSS exhibiting geographic distribution which strongly correlated with the mtDNA divergence in chameleons across the Jezreel Valley. As our previous study excluded mtDNA introgression, which is the most common explanation for mito-nuclear discordance (Toews and Brelsford 2012), and because there was no apparent sex bias in the dispersal of the two chameleon mtDNA types, two explanations remained: 1) certain mito-nuclear genotype combinations are better adapted to the north or to the southern part of the Jezreel Valley and 2) the north and south mito-nuclear genotype combinations represent compensatory responses to functional mtDNA changes that separately accumulated in the two chameleon groups during the long-term existence of the ancient marine barrier. Because there is no prominent environmental difference between the northern and southern parts of the valley, the first explanation is unlikely to account for the sharp north–south mtDNA divergence. Our mathematical formulation was consistent with the second explanation: The model indicated that the sharp north–south mtDNA differentiation could be explained by the breakdown of crucial mito-nuclear interactions, especially when hybrid females mate with a male with the opposite mtDNA haplotype. Therefore, similar to previous observations in copepods (Burton and Barreto 2012), our findings imply that interfering with mito-nuclear genotype compatibility in chameleons likely leads to hybrid breakdown. Hybrid breakdown alludes to the formation of reproductive barriers, the first requirement preceding speciation events. Hence, mito-nuclear gene flow arrest between chameleons across the Jezreel Valley likely reflects incipient allopatric speciation event.

Our findings indicate that of the 14 chameleon orthologs that were identified as candidates to correlate with the mtDNA north–south divergence, only 6 genes withstood the north–south differentiation in the analysis of an enlarged chameleon sample size. Strikingly, these six proteins have known interactions with mtDNA-encoded components: *POLRMT* codes for the mitochondrial RNA polymerase which interacts with the mtDNA promoter (Gaspari et al. 2004); *NDUFA5* encodes for one of the subunits in complex 1 of the OXPHOS system, which could interact with one of the seven mtDNA-encoded complex I subunits; *ACO1* encodes for cytoplasmic aconitase hydratase that is also localized to the mitochondria, which probably interacts with the mtDNA, and was shown to be important for mtDNA stability (Chen et al. 2005, 2007); *LYRM4* codes for a protein having functional importance for the respiratory chain complexes (Lim et al. 2013); MARS2 codes for the mitochondrial methionine tRNA synthetase which tethers methionine to the mtDNA-encoded tRNA; notably, because no north–south diverging mutations were identified in the corresponding mtDNA tRNA^Met^_,_ compensatory mechanism may be less likely. Finally, *ACAD9* codes for one of the assembly factors of OXPHOS complex 1 (Haack et al. 2010). This evidence further suggests that mito-nuclear interactions could underlie, at least in part, the inferred north–south hybrid breakdown in chameleons.

If such population divergence indeed marked the functional incompatibility between the nDNA and mtDNA from the north and south populations, one would expect that the mito-nuclear genotype combinations which underlie this divergence will comprise mutations with clear functional differences. Indeed, our functionality assessment revealed that mutations in some of the identified transcripts which differentiate the south and north populations alter amino acid with high functionality or structural implications. Two mutations exemplify our argument: 1) the mutation that occurred at the DNA binding domain of the mitochondrial RNA polymerase (POLRMT) and 2) the positively selected mutation in NDUFA5. Although considering the POLRMT mutation, many of the fixed mtDNA mutations that differentiate the north from south chameleons occurred within the putative promoters region. One could envision possible mito-nuclear incompatibility in the activation of northern promoters by southern POLRMT and vice versa. This possibility is supported by the observation that human POLRMT cannot activate transcription of a mouse mtDNA promoter (Gaspari et al. 2004). Moreover, POLRMT sequence differences played a major role in alterations of mtDNA gene expression and copy number in intrapopulation crosses of copepods (Ellison and Burton 2008, 2010), further suggesting population-specific mito-nuclear interactions. Second, it is tempting to suggest that the positively selected amino acid in NDUFA5 that divided the south and north chameleon populations reflect an evolutionary response to mtDNA changes in complex I. Indeed, our correlated mutations test highlighted ND5 as the most plausible coevolving partner of NDUFA5, further supporting mito-nuclear coevolution in chameleons.

If incipient speciation explains our findings, could mito-nuclear epistatic incompatibility solely underlie this event? As many of the 190 gene candidates that separate the north from the south chameleon populations do not have a clear mitochondrial function, we cannot exclude the involvement of additional mechanisms. Nevertheless, none of these genes constitute good candidates to clearly explain our previously observed sharp mtDNA divergence between the north and south chameleon populations. Theodosius Dobzhansky and Herman Joseph Muller separately suggested almost 80 years ago that it is sufficient to interfere with a single epistatic interaction to trigger a speciation event (Dobzhansky 1936; Muller 1942). Therefore, only a single pair of mito-nuclear genes is sufficient (let alone six that we identified) to genetically diverge the two chameleon populations and persevere the mtDNA geographic pattern of distribution. A recent study in sparrows suggested that at least in the case of the Italian and Spanish sparrows mitochondrial and sex linked genes are deeply involved in hybrid speciation (Trier et al. 2014). In this study, we do not argue for hybrid speciation, but rather for an incipient speciation process, commenced as an allopatric event due to geographic separation by an ancient marine barrier (currently the Jezreel Valley). The marked paucity of hybrids carrying a certain nDNA genotype with either mtDNA type (north or south) supports the true existence of a reproductive barrier among morphologically indistinguishable chameleons. In order to experimentally test whether the putative reproductive barrier is pre- or postzygotic, reciprocal mating experiments should be performed between individuals from the south and the north for at least two generations. *Chamaeleo chamaeleon recticrista* individuals live around 2 years in nature, mate only once a year, and their eggs hatch only the year after. This poses a major experimental obstacle. However, the molecular compatibility of our discovered nDNA and mtDNA genotypes could be tested in cells or in vitro. This line of thought guides us for the design of our future steps.

We noticed that among the 335 fixed mtDNA mutations that differentiated the two chameleon populations, 63 were NSSs, 23 were in RNA genes, and the rest were either synonymous or within the control noncoding region. Our functional and structural analyses clearly indicated high functional potential of a subset of the observed amino acid changes, especially in complex I subunits. Along with this finding, one may argue that many of the fixed mutations have limited potential function which simply reflects the time of divergence between the studied populations. Nevertheless, one cannot dispose all synonymous and noncoding mutations as nonfunctional, because at least a subset of such mutations may alter regulatory elements including promoters, repressors, and enhancers. Moreover, regulatory elements have been described within protein-coding sequences, thus implying dual roles for synonymous mutations—they may both alter codon usage and have regulatory impact (Birnbaum et al. 2012; Stergachis et al. 2013). Recently, our group showed that mtDNA binding by bona fide transcription factors may occur within protein-coding genes thus further supporting such a possibility in the mtDNA (Blumberg et al. 2014). Taken together, our analyses provide candidates for mito-nuclear coevolution that associate with chameleon population divergence in Israel.

In summary, our results underline mito-nuclear genetic interaction as an attractive candidate mechanism involved in the formation of reproductive barriers and subsequent speciation event in vertebrates.

## Data Access

The RNA-seq data of the first chameleon^30^ can be accessed through the Sequence Reads Archive (http://www.ncbi.nlm.nih.gov/sra, last accessed December 1, 2015), accession number SRP029972. The other nine RNA-seq data sets have been submitted to SRA and were given the following accession numbers: Study accession: SRP066727–PRJNA301500; Runs accessions: SRR2962870–SRR2962878.

## Supplementary Material

Supplementary file S1, figures S1 and S2, and tables S1–S12 are available at *Genome Biology and Evolution* online (http://www.gbe.oxfordjournals.org/).

Supplementary Data
