# Supplementary material for: Mitochondrial Involvement in Vertebrate Speciation? The Case of Mito-nuclear Genetic Divergence in Chameleons
Source: Genome Biol Evol. 2015 Nov 19;7(12):3322–36. doi: 10.1093/gbe/evv226 (PMC4700957; doi:10.1093/gbe/evv226)
Supplement: Supplementary Data [file supp_evv226_suppl_data.zip › BarYaacov2015_Chameleons_SupplementaryTable10.docx]

| **Gene** | **Reaction mix** | **PCR conditions** | **PCR fragment length** | **Amplification primers** | **Sequencing primers** |
| --- | --- | --- | --- | --- | --- |
| *POLRMT* 1090 | Phusion | 98°C for 5 min, followed by 35 cycles including denaturation (98°C, 30 sec), annealing (70°C, 20 sec) and elongation (72°C, 3.5 min). The cycles were followed by a final extension step (72°C, 7 min). | 3500 bp | 1 (Forward) and 2 (Reverse) | 3 |
| *POLRMT* 1090 | Taq | 94°C for 5 min, followed by 35 cycles including denaturation (94°C, 30 sec), annealing (55°C, 30 sec) and elongation (72°C, 20 sec). The cycles were followed by a final extension step (72°C, 7 min). | 200 bp | 1 (Forward) and 3 (Reverse) | 1 and 3 |
| *POLRMT* 1218 | Taq | 94°C for 5 min, followed by 35 cycles including denaturation (94°C, 30 sec), annealing (54°C, 30 sec) and elongation (72°C, 3 min). The cycles were followed by a final extension step (72°C, 7 min) | 2500 bp | 4 (Forward) and 5 (Reverse) | 5 |
| *POLRMT* 1218 | Phusion | 98°C for 5 min, followed by 35 cycles including denaturation (98°C, 30 sec), annealing (61°C, 30 sec) and elongation (72°C, 20 sec). The cycles were followed by a final extension step (72°C, 7 min). | 252 bp | 6 (Forward) and 7 (Reverse) | RFLP |
| *SDHC* | Phusion | 98°C for 5 min, followed by 35 cycles including denaturation (98°C, 30 sec), annealing (67°C, 20 sec) and elongation (72°C, 4 min). The cycles were followed by a final extension step (72°C, 7 min) | 4000 bp | 8 (Forward) and 9 (Reverse) | 8 |
| *SDHC* | Taq | 94°C for 5 min, followed by 35 cycles including denaturation (94°C, 30 sec), annealing (58°C, 30 sec) and elongation (72°C, 30 sec). The cycles were followed by a final extension step (72°C, 7 min) | 400 bp | 8 (Forward) and 10 (Reverse) | 10 |
| *MARS2* | Taq | 94°C for 5 min, followed by 35 cycles including denaturation (94°C, 30 sec), annealing (55°C, 30 sec) and elongation (72°C, 60 sec). The cycles were followed by a final extension step (72°C, 7 min) | 800 bp | 11 (Forward) and 12 (Reverse) | 11 |
| *NDUFA5* | Taq | 94°C for 5 min, followed by 35 cycles including denaturation (94°C, 30 sec), annealing (60°C, 30 sec) and elongation (72°C, 60 sec). The cycles were followed by a final extension step (72°C, 7 min) | 850 bp | 13 (Forward) and 14 (Reverse) | 15 |
| *MRPL30* | Taq | 94°C for 5 min, followed by 35 cycles including denaturation (94°C, 30 sec), annealing (60°C, 30 sec) and elongation (72°C, 2 min). The cycles were followed by a final extension step (72°C, 7 min) | 2000 bp | 16 (Forward) and 17 (Reverse) | 16 |
| *MRPL30* | Taq | 94°C for 5 min, followed by 35 cycles including denaturation (94°C, 30 sec), annealing (69.4°C, 30 sec) and elongation (72°C, 30 sec). The cycles were followed by a final extension step (72°C, 7 min) | 250 bp | 16 (Forward) and 18 (Reverse) | 18 and RFLP |
| *ACAD9* | Taq | 94°C for 5 min, followed by 35 cycles including denaturation (94°C, 30 sec), annealing (55°C, 30 sec) and elongation (72°C, 1 min). The cycles were followed by a final extension step (72°C, 7 min) | 764 bp | 19 (Forward) and 20 (Reverse) | 19, 21 and 22 |
| *TCIRG1* | Taq | 94°C for 5 min, followed by 35 cycles including denaturation (94°C, 30 sec), annealing (55°C, 30 sec) and elongation (72°C, 15 sec). The cycles were followed by a final extension step (72°C, 7 min) | 161 bp | 23 (Forward) and 24 (Reverse) | 23 |
| *AARS2* | Taq | 94°C for 5 min, followed by 35 cycles including denaturation (94°C, 30 sec), annealing (61°C, 30 sec) and elongation (72°C, 2.5 min). The cycles were followed by a final extension step (72°C, 7 min) | 2000 bp | 25 (Forward) and 26 (Reverse) | 25 |
| *AARS2* | Taq | 94°C for 5 min, followed by 35 cycles including denaturation (94°C, 30 sec), annealing (66°C, 30 sec) and elongation (72°C, 12 sec). The cycles were followed by a final extension step (72°C, 7 min) | 118 bp | 27 (Forward) and 26 (Reverse) | 26 |
| *C1QBP (P32)* | Taq | 94°C for 5 min, followed by 35 cycles including denaturation (94°C, 30 sec), annealing (60°C, 30 sec) and elongation (72°C, 12 sec). The cycles were followed by a final extension step (72°C, 7 min) | 125 bp | 28 (Forward) and 29 (Reverse) | 29 |
| *ACO1* | Taq | 94°C for 5 min, followed by 35 cycles including denaturation (94°C, 30 sec), annealing (53°C, 30 sec) and elongation (72°C, 12 sec). The cycles were followed by a final extension step (72°C, 7 min) | 116 bp | 30 (Forward) and 31 (Reverse) | 30 |
| *ETFA* | Taq | 94°C for 5 min, followed by 35 cycles including denaturation (94°C, 30 sec), annealing (66°C, 30 sec) and elongation (72°C, 15 sec). The cycles were followed by a final extension step (72°C, 7 min) | 206 bp | 32 (Forward) and 33 (Reverse) | 34 |
| *LYRM4* | Taq | 94°C for 5 min, followed by 35 cycles including denaturation (94°C, 30 sec), annealing (55°C, 30 sec) and elongation (72°C, 15 sec). The cycles were followed by a final extension step (72°C, 7 min) | 190 bp | 35 (Forward) and 36 (Reverse) | 35 |
| *LYRM4* | Taq | 94°C for 5 min, followed by 35 cycles including denaturation (94°C, 30 sec), annealing (59°C, 30 sec) and elongation (72°C, 10 sec). The cycles were followed by a final extension step (72°C, 7 min) | 100 bp | 37 (Forward) and 38 (Reverse) | RFLP |
