# Supplementary material for: Mitochondrial Involvement in Vertebrate Speciation? The Case of Mito-nuclear Genetic Divergence in Chameleons
Source: Genome Biol Evol. 2015 Nov 19;7(12):3322–36. doi: 10.1093/gbe/evv226 (PMC4700957; doi:10.1093/gbe/evv226)
Supplement: Supplementary Data [file supp_evv226_suppl_data.zip › BarYaacov2015_Chameleons_SupplementaryTable11.docx]

| **Gene** | **Enzyme** | **Reaction mix** | **Restriction conditions** |
| --- | --- | --- | --- |
| *POLRMT* 1218 | Taq^α^I #R0149S | 0.5 µl (10 units) of Taq^α^I, 2 µl of “CutSmart” buffer mix (10X), 8 µl of PCR reaction; 9.5 µl of double distilled water (DDW) were added to a total volume of 20 µl | 65°C for 30 minutes, following an inactivation step of 10 minutes in 80°C |
| *POLRMT* 1218 | MlucI #R0538S | 1 µl (10 units) of MlucI, 2 µl of “CutSmart” buffer mix (10X), 8 µl of PCR reaction; 9 µl of double distilled water (DDW) were added to a total volume of 20 µl | 37°C for 30 minutes, followed by an inactivation step of 10 minutes in 80°C |
| *MRPL30* | PstI #R0140S | 0.5 µl (10 units) of PstI, 2 µl of “3.1” buffer mix (10X), 8 µl of PCR reaction; 9.5 µl of DDW were added to a total volume of 20 µl | 37°C for 30 minutes, following an inactivation step of 10 minutes in 80°C |
| *ETFA* | HindIII #ER0501 | 1 µl (10 units) of HindIII, 2 µl of “red” buffer mix (10X), 8 µl of PCR reaction; 9 µl of DDW were added to a total volume of 20 µl | 37°C for 4 hours, followed by an inactivation step of 10 minutes in 80 °C |
| *LYRM4* | ApekI #R0643S | 1 µl (5 units) of ApekI, 2 µl of “3.1” buffer mix (10X), 10 µl of PCR reaction; 7 µl of DDW were added to a total volume of 20 µl | 75 °C for 60 minutes |
