# Supplementary material for: Mitochondrial Involvement in Vertebrate Speciation? The Case of Mito-nuclear Genetic Divergence in Chameleons
Source: Genome Biol Evol. 2015 Nov 19;7(12):3322–36. doi: 10.1093/gbe/evv226 (PMC4700957; doi:10.1093/gbe/evv226)
Supplement: Supplementary Data [file supp_evv226_suppl_data.zip › BarYaacov2015_Chameleons_Supplementary methods.docx]

**Supplementary methods – mathematical model**

We alter the mathematical formulation in the main text to consider loci that are polymorphic. We assume that the north population, *P_N_*, is polymorphic at *n* nDNA loci interacting with its mitochondria so that at the *i*^th^ locus we observe (*a_i_a*_i_, *A_i_a*_i_, *A_i_A*_i_) at ratio 1:2:1. The south population, *P_S_*, has different mitochondria that require a homozygote at each locus. Without loss of generality we can assume this is *a_i_a*_i_, for locus *i*. As individuals carrying *m_N_* mitochondria have high fitness for all genotypes we only consider fitness impairment in individuals with *m_S_* mitochondria. As in the main text, we will consider the mean fitness for F1, F2 and F1 backcrosses. To make the derivation clearer, Table S1 provides the expected frequency of each genotype *a_i_a*_i_, *A_i_a*_i_ and *A_i_A*_i_ at locus *i*. This is independent of *i* as we assume the same pattern for all loci and no linkage between them. We further assume that heterozygotes with the *m_S_* mitochondria have fitness 1- *s*_1_ and homozygotes AA with the *m_S_* mitochondria have fitness 1- *s*_2_. Based on these assumptions and the frequencies in Table 1, we can derive the expected fitness for F1, F2 and backcrosses as follows. The expected fitness of F1 individuals is given by (Equation S1):

$$W_{1}=\sum_{i=0}^{n} \left(\begin{matrix} n \\ i \end{matrix}\right)\left(\frac{1}{2}\right)^{n}(1-s_{1})^{i}$$

where the sum is over *i*, the number of heterozygotes in an F1 individual. The values of *s*_1_ can be very small or zero, for example due to dominance of the co-adapted allele. The expected fitness for F2 individuals is given by (Equation S2),

$$\begin{matrix} W_{2}=\sum_{j=0}^{n-i} \sum_{i=0}^{n} P(x=i,y=j,z=n-i-j)(1-s_{2})^{i}(1-s_{1})^{j} \\ =\sum_{j=0}^{n-i} \sum_{i=0}^{n} \left(\frac{n!}{i!j!(n-i-j)!}\right)\left(\frac{1}{16}\right)^{i}\left(\frac{6}{16}\right)^{j}\left(\frac{9}{16}\right)^{n-i-j}(1-s_{2})^{i}(1-s_{1})^{j} \end{matrix}$$

where *x* is the number of the homozygotes mismatched to the mitochondria of the zygote, *y* is the number of heterozygotes and *z* is the number of homozygotes that match the mitochondria background. The expected fitness of F1 males backcrossed to *P_S_* and F1 females with the south mitochondria backcrossed to *P_S_*, is given by (Equation S3),

$$\begin{matrix} W_{3}=\sum_{i=0}^{n} P(x=i,y=n-j)(1-s_{1})^{i} \\ =\sum_{i=0}^{n} \left(\begin{matrix} n \\ i \end{matrix}\right)\left(\frac{1}{4}\right)^{i}\left(\frac{3}{4}\right)^{n-1}(1-s_{1})^{i} \end{matrix}$$

where *x* is the number of heterozygote loci and *y* is the number of homozygotes matching the south mitochondria background. Finally, the expected fitness of females with *m_S_* mitochondria backcrossed to parental *P_N_* males, is given by (Equation S4),

$$\begin{matrix} W_{4}=\sum_{j=0}^{n-i} \sum_{i=0}^{n} P(x=i,y=j,z=n-i-j)(1-s_{2})^{i}(1-s_{1})^{j} \\ =\sum_{j=0}^{n-i} \sum_{i=0}^{n} \left(\frac{n!}{i!j!(n-i-j)!}\right)\left(\frac{1}{8}\right)^{i}\left(\frac{4}{8}\right)^{j}\left(\frac{3}{8}\right)^{n-i-j}(1-s_{2})^{i}(1-s_{1})^{j} \end{matrix}$$

where *x* is the number of homozygotes matching *m_S_*, *y* is the number of heterozygote loci and *z* is the number of homozygotes not matching *m_S_*.

We plotted *W_1_*-*W_4_* on Supplementary Fig. 1. This indicates that a very similar pattern to that observed when assuming homozygote loci only as in the main text. However, fitness reduction due to mito-nuclear mismatch is now less steep. Variation on the cost for heterozygotes and the wrong homozygotes in the south population can result in more or less severe reproductive barriers between the two populations.

Table S1: Expected genotype frequencies for F1 and F2 hybrids, and F1 backcrosses. We only consider crosses that will result in *m_S_* mitochondria.

|  | *aa* | *Aa* | *AA* |
| --- | --- | --- | --- |
| F1 | 1/2 | 1/2 | 0 |
| F2 | 9/16 | 6/16 | 1/16 |
| F1 backcross 1* | 3/4 | 1/4 | 0 |
| F1 backcross 2** | 3/8 | 1/2 | 1/8 |

*F1 males and F1 females with the south mitochondria backcrossed to *P_S_* **F1 females with the south mitochondrial backcrossed to *P_N_*
