# Supplementary material for: Mitochondrial Involvement in Vertebrate Speciation? The Case of Mito-nuclear Genetic Divergence in Chameleons
Source: Genome Biol Evol. 2015 Nov 19;7(12):3322–36. doi: 10.1093/gbe/evv226 (PMC4700957; doi:10.1093/gbe/evv226)
Supplement: Supplementary Data [file supp_evv226_suppl_data.zip › BarYaacov2015_Chameleons_SupplementaryTable1.docx]

| **Sample** | **Location** | **Sample** | **Location** |
| --- | --- | --- | --- |
| 69 | Ha’on | 66 | Kerem Maharal |
| 71 | Ha’on | 75 | Carmel |
| 77 | Akbara | 105 | Habonim |
| 81 | Akbara | 106 | Habonim |
| 86 | Korazim | 108 | Jerusalem |
| 88 | Korazim | 112 | Caesarea |
| 90 | Korazim | 129 | Kishon |
| 94 | Ramot | 130 | Kiryat Haroshet |
| 97 | Poria | 131 | Kiryat Haroshet |
| 99 | Shamir | 133 | Kiryat Haroshet |
| 101 | Shamir | 136 | Mt. Gahar |
| 102 | Shamir | 137 | Mt. Gahar |
| 113 | Bar’am | 139 | Mt. Gahar |
| 114 | Bar’am | 150 | Salem |
| 115 | Fasuta | 151 | Salem |
| 116 | Fasuta | 156 | Megido junction |
| 123 | Magen Shaul | 157 | Gadish |
| k121 | Magen Shaul | 164 | Tivon |
| K122 | Magen Shaul | 165 | Tivon |
| K125 | Beit Hashita | 169 | Usha |
| D1 | Bar’am forest entry | D6 | Eliakim junction - 2 km west to the junction on the road towards Daliat El Carmel |
| D2 | Road to Meron field school | D7 | On the ROAD to Meir Shfeia school |
| D3 | Between Neve Ziv and Maalot – on road 89 | D8 | Close to the Electric company road, near Or-Akiva |
| D4 | KKL parking lot next to Neve Ziv | D9 | Habonim |
| D5 | Cemetery next to Horvat Turit, on road 85. | D13 | Hazorea |
| D10 | Kfar Hananya | D14 | Mishmar HaEmek |
| D11 | Karmiel | D15 | Givaat Oz |
| D12 | Yodfat | D16 | Yiron forest next to entrance to road 6 from road 65 |
| D17 | Alon Hagalil | D24 | Road 651 next to Caesarea |
| D18 | Hoshaya forest | D25 | Road 65 west next to Hadera Power station |
| D19 | Beit Rimon | D26 | Hadera |
| D20 | Mitzpe Netofa | D27 | Mikhmoret |
| D21 | Havat Hashomer | D28 | Havatzelet Hasharon |
| D22 | Beit Keshet | D29 | Road 5611 - South of Netanya |
| D23 | Shibly | D30 | Herzliya Marine |
