# Supplementary material for: Mitochondrial Involvement in Vertebrate Speciation? The Case of Mito-nuclear Genetic Divergence in Chameleons
Source: Genome Biol Evol. 2015 Nov 19;7(12):3322–36. doi: 10.1093/gbe/evv226 (PMC4700957; doi:10.1093/gbe/evv226)
Supplement: Supplementary Data [file supp_evv226_suppl_data.zip › BarYaacov2015_Chameleons_SupplementaryTable3.docx]

| **Category\Sample ID** | **D1** | **D2** | **D4** | **D6** | **D7** | **D8** | **D10** | **D12** | **D13** | **D16** |
| --- | --- | --- | --- | --- | --- | --- | --- | --- | --- | --- |
| Q1 (bp) | 464 | 450 | 480 | 456 | 458 | 461 | 452 | 451 | 453 | 450 |
| Med (bp) | 1278 | 1203 | 1407 | 1228 | 1292 | 1281 | 1279 | 1306 | 1268 | 1273 |
| Q3 (bp) | 2899 | 2875 | 3156 | 2717 | 3033 | 3006 | 3184 | 3165 | 3047 | 3096 |
| Min (bp) | 200 | 200 | 200 | 200 | 200 | 200 | 200 | 200 | 200 | 200 |
| Max (bp) | 17251 | 21856 | 21956 | 16291 | 23157 | 20537 | 24323 | 23487 | 32772 | 22571 |
| Mean (bp) | 753 | 736 | 785 | 741 | 753 | 755 | 750 | 751 | 749 | 746 |
| Total | 68068 | 76423 | 68067 | 63669 | 72746 | 80125 | 79208 | 83519 | 69236 | 75087 |
