# Supplementary material for: Mitochondrial Involvement in Vertebrate Speciation? The Case of Mito-nuclear Genetic Divergence in Chameleons
Source: Genome Biol Evol. 2015 Nov 19;7(12):3322–36. doi: 10.1093/gbe/evv226 (PMC4700957; doi:10.1093/gbe/evv226)
Supplement: Supplementary Data [file supp_evv226_suppl_data.zip › BarYaacov2015_Chameleons_SupplementaryTable5.docx]

| Gene/Protein | Number of mutations |
| --- | --- |
| gi_110618253\|ref_NP_005026.3\|DNA-directed RNA polymerase mitochondrial precursor | 2 |
| gi_21361497\|ref_NP_054768.2\|acyl-CoA dehydrogenase family member 9 mitochondrial | 1 |
| gi_24308436\|ref_NP_612404.1\|methionine--tRNA ligase mitochondrial precursor | 3 |
| gi_4826848\|ref_NP_004991.1\|NADH dehydrogenase [ubiquinone] 1 alpha subcomplex subunit 5 | 1 |
| gi_8659555\|ref_NP_002188.1\|cytoplasmic aconitate hydratase | 1 |
| gi_19924145\|ref_NP_006010.2\|V-type proton ATPase 116 kDa subunit a isoform 3 isoform a | 1 |
| gi_21735604\|ref_NP_660213.1\|39S ribosomal protein L30 mitochondrial precursor | 1 |
| gi_38569417\|ref_NP_065796.1\|alanine--tRNA ligase mitochondrial | 1 |
| gi_4502491\|ref_NP_001203.1\|complement component 1 Q subcomponent-binding protein mitochondrial precursor | 1 |
| gi_4503607\|ref_NP_000117.1\|electron transfer flavoprotein subunit alpha mitochondrial isoform a | 1 |
| gi_4506863\|ref_NP_002992.1\|succinate dehydrogenase cytochrome b560 subunit mitochondrial isoform 1 precursor | 1 |
| gi_9665248\|ref_NP_000584.2\|antigen peptide transporter 1 | 1 |
| gi_38570054\|ref_NP_065141.3\|LYR motif-containing protein 4 isoform 1 | 1 |
| gi_5453884\|ref_NP_006205.1\|phytanoyl-CoA dioxygenase peroxisomal isoform a precursor | 2 |
| gi_108773810\|ref_NP_064502.9\|leucine--tRNA ligase cytoplasmic | 2 |
| gi_108796666\|ref_NP_001004301.2\|zinc finger protein 813 | 1 |
| gi_109715835\|ref_NP_071353.4\|HEAT repeat-containing protein 6 | 1 |
| gi_109948283\|ref_NP_055693.4\|pumilio domain-containing protein KIAA0020 | 1 |
| gi_110349778\|ref_NP_066971.2\|zinc finger protein 273 | 1 |
| gi_110626177\|ref_NP_940937.1\|zinc finger protein 699 | 1 |
| gi_110815802\|ref_NP_115683.3\|UPF0553 protein C9orf64 | 1 |
| gi_112983683\|ref_NP_689688.2\|zinc finger protein 417 | 1 |
| gi_11386193\|ref_NP_008922.1\|zinc finger protein 197 isoform 1 | 1 |
| gi_116008442\|ref_NP_055885.3\|zinc finger CCCH domain-containing protein 13 | 2 |
| gi_116063554\|ref_NP_057038.2\|putative deoxyribose-phosphate aldolase | 1 |
| gi_116235451\|ref_NP_997226.2\|RING finger protein 214 isoform 1 | 1 |
| gi_117320527\|ref_NP_002493.3\|nuclear factor NF-kappa-B p100 subunit isoform b | 1 |
| gi_119226229\|ref_NP_710155.2\|zinc finger protein 1 homolog | 1 |
| gi_119360348\|ref_NP_000138.2\|tissue alpha-L-fucosidase precursor | 1 |
| gi_12007648\|ref_NP_072090.1\|aldehyde dehydrogenase family 8 member A1 isoform 1 | 2 |
| gi_122114654\|ref_NP_076991.3\|methyltransferase-like protein 16 | 1 |
| gi_13236528\|ref_NP_077277.1\|fukutin-related protein | 6 |
| gi_13378137\|ref_NP_003780.1\|tumor necrosis factor receptor type 1-associated DEATH domain protein | 1 |
| gi_138175821\|ref_NP_037512.3\|zinc finger protein 112 isoform 2 | 1 |
| gi_13994151\|ref_NP_066272.1\|PDZ and LIM domain protein 1 | 1 |
| gi_14141195\|ref_NP_008854.2\|stromal cell-derived factor 2 precursor | 1 |
| gi_14249456\|ref_NP_116177.1\|zinc finger protein 514 | 1 |
| gi_14269566\|ref_NP_065882.1\|MKL/myocardin-like protein 1 | 1 |
| gi_146094506\|ref_NP_001078868.1\|RELT-like protein 1 precursor | 1 |
| gi_146134388\|ref_NP_062535.2\|YLP motif-containing protein 1 | 1 |
| gi_147905620\|ref_NP_064612.2\|histone-lysine N-methyltransferase PRDM9 | 3 |
| gi_148612893\|ref_NP_001091961.1\|zinc finger protein 419 isoform 1 | 1 |
| gi_148839292\|ref_NP_078800.3\|probable cation-transporting ATPase 13A3 | 1 |
| gi_149589008\|ref_NP_000276.2\|xaa-Pro dipeptidase isoform 1 | 1 |
| gi_152963635\|ref_NP_001092908.1\|zinc finger protein 30 isoform a | 3 |
| gi_152963637\|ref_NP_001002836.2\|zinc finger protein 787 | 2 |
| gi_153792407\|ref_NP_612376.1\|zinc finger protein 251 | 1 |
| gi_154091003\|ref_NP_001001668.3\|zinc finger protein 470 | 1 |
| gi_154146262\|ref_NP_003881.2\|IgGFc-binding protein precursor | 3 |
| gi_154350236\|ref_NP_062546.2\|protein FAM214A | 1 |
| gi_156231355\|ref_NP_110386.2\|nuclear receptor-binding factor 2 | 1 |
| gi_157388995\|ref_NP_060727.2\|protein-L-isoaspartate O-methyltransferase domain-containing protein 2 isoform 1 | 1 |
| gi_157419154\|ref_NP_002996.2\|P-selectin precursor | 1 |
| gi_157694503\|ref_NP_009094.3\|urea transporter 2 | 1 |
| gi_16418467\|ref_NP_443204.1\|leucine-rich alpha-2-glycoprotein precursor | 1 |
| gi_164419743\|ref_NP_001104792.1\|ATP-dependent RNA helicase DDX54 isoform 1 | 3 |
| gi_166197658\|ref_NP_001723.2\|butyrophilin subfamily 1 member A1 precursor | 2 |
| gi_166235163\|ref_NP_036477.2\|nuclear fragile X mental retardation-interacting protein 1 | 1 |
| gi_166795297\|ref_NP_057225.2\|cytoplasmic dynein 1 light intermediate chain 1 | 1 |
| gi_17738285\|ref_NP_061983.2\|zinc finger protein with KRAB and SCAN domains 4 | 1 |
| gi_17864094\|ref_NP_064562.1\|protein fem-1 homolog C | 1 |
| gi_18087821\|ref_NP_542387.1\|CDKN2AIP N-terminal-like protein | 1 |
| gi_186910296\|ref_NP_001119574.1\|haptoglobin isoform 2 preproprotein | 2 |
| gi_186928839\|ref_NP_689656.2\|retinol dehydrogenase 12 precursor | 1 |
| gi_187607437\|ref_NP_056495.3\|protein CNPPD1 | 2 |
| gi_188528633\|ref_NP_009066.2\|zinc finger protein 79 | 2 |
| gi_190014599\|ref_NP_001121695.1\|zinc finger protein 717 | 4 |
| gi_190014620\|ref_NP_597721.2\|zinc finger protein 483 isoform a | 1 |
| gi_190358543\|ref_NP_065945.2\|UV-stimulated scaffold protein A | 1 |
| gi_194097365\|ref_NP_060117.3\|pre-rRNA processing protein FTSJ3 | 2 |
| gi_194097392\|ref_NP_004477.3\|Golgin subfamily A member 2 | 1 |
| gi_197384097\|ref_NP_001128128.1\|zinc finger protein 662 isoform 2 | 1 |
| gi_19923951\|ref_NP_612434.1\|protein C10 | 1 |
| gi_20149619\|ref_NP_056325.2\|dehydrogenase/reductase SDR family member 7B | 1 |
| gi_207452735\|ref_NP_112598.2\|epiplakin | 1 |
| gi_21264343\|ref_NP_002958.2\|scaffold attachment factor B1 isoform 3 | 1 |
| gi_21327667\|ref_NP_056016.1\|ribosome biogenesis protein BOP1 | 1 |
| gi_21359865\|ref_NP_004248.2\|transforming growth factor-beta receptor-associated protein 1 | 1 |
| gi_21361274\|ref_NP_006007.2\|sialomucin core protein 24 isoform 1 precursor | 2 |
| gi_21361886\|ref_NP_116238.2\|mesenteric estrogen-dependent adipogenesis protein | 1 |
| gi_21630277\|ref_NP_660215.1\|E3 ubiquitin-protein ligase TRIM11 | 2 |
| gi_217272865\|ref_NP_004678.3\|myotubularin-related protein 4 | 2 |
| gi_221136858\|ref_NP_001137462.1\|telomerase Cajal body protein 1 | 1 |
| gi_221316749\|ref_NP_001861.2\|mast cell carboxypeptidase A precursor | 1 |
| gi_223029512\|ref_NP_001138554.1\|zinc finger protein 619 isoform 1 | 1 |
| gi_22325377\|ref_NP_003822.2\|serine/threonine-protein kinase RIO3 | 1 |
| gi_224809432\|ref_NP_861448.2\|transmembrane and TPR repeat-containing protein 3 | 2 |
| gi_22547224\|ref_NP_003763.2\|jerky protein homolog-like | 4 |
| gi_226498382\|ref_NP_612385.2\|tetratricopeptide repeat protein 5 | 1 |
| gi_22749235\|ref_NP_689814.1\|zinc finger protein 709 | 1 |
| gi_23097323\|ref_NP_689839.1\|zinc finger protein 92 isoform 2 | 1 |
| gi_23510356\|ref_NP_113668.2\|serine/threonine-protein kinase RIO1 isoform 1 | 1 |
| gi_241666479\|ref_NP_112169.2\|REM2- and Rab-like small GTPase 1 | 2 |
| gi_24307991\|ref_NP_055904.1\|cullin-9 | 1 |
| gi_24308039\|ref_NP_056138.1\|E3 UFM1-protein ligase 1 | 1 |
| gi_24430146\|ref_NP_005115.2\|nuclear pore complex protein Nup153 isoform 2 | 1 |
| gi_24431977\|ref_NP_060523.2\|XK-related protein 8 | 1 |
| gi_24797076\|ref_NP_002112.3\|HLA class II histocompatibility antigen DP beta 1 chain precursor | 1 |
| gi_257470984\|ref_NP_003427.3\|zinc finger protein 135 isoform 2 | 6 |
| gi_257900512\|ref_NP_073581.2\|UPF0420 protein C16orf58 | 1 |
| gi_26080431\|ref_NP_079133.3\|ATPase family AAA domain-containing protein 5 | 1 |
| gi_269315880\|ref_NP_115554.2\|probable RNA polymerase II nuclear localization protein SLC7A6OS | 1 |
| gi_27734761\|ref_NP_775956.1\|E3 SUMO-protein ligase NSE2 | 1 |
| gi_27734905\|ref_NP_775816.1\|aprataxin and PNK-like factor | 1 |
| gi_283436133\|ref_NP_001164433.1\|immunoglobulin superfamily member 1 isoform 4 | 1 |
| gi_28626504\|ref_NP_113659.3\|fermitin family homolog 3 short form | 1 |
| gi_28626521\|ref_NP_066363.1\|NFX1-type zinc finger-containing protein 1 | 2 |
| gi_291219924\|ref_NP_938146.1\|signal transducer and activator of transcription 2 isoform 2 | 1 |
| gi_296011010\|ref_NP_060252.4\|protein FAM208B | 1 |
| gi_29826282\|ref_NP_817092.1\|protein phosphatase 1G | 1 |
| gi_30425510\|ref_NP_848642.1\|protein archease | 1 |
| gi_305682571\|ref_NP_008900.3\|zinc finger protein 28 | 1 |
| gi_307078123\|ref_NP_001182484.1\|clathrin interactor 1 isoform 1 | 1 |
| gi_30794216\|ref_NP_112223.1\|E3 ubiquitin-protein ligase TRIM56 | 1 |
| gi_31543983\|ref_NP_115765.2\|ADP-ribosylation factor GTPase-activating protein 2 isoform 1 | 1 |
| gi_32454746\|ref_NP_859525.1\|origin recognition complex subunit 4 isoform 1 | 1 |
| gi_32526896\|ref_NP_056211.2\|armadillo repeat-containing protein 8 isoform 2 | 1 |
| gi_33188445\|ref_NP_036222.3\|microtubule-actin cross-linking factor 1 | 2 |
| gi_33342268\|ref_NP_620129.2\|R3H domain-containing protein 4 | 1 |
| gi_339276021\|ref_NP_001229824.1\|zinc finger protein with KRAB and SCAN domains 3 isoform 2 | 1 |
| gi_345842380\|ref_NP_001230967.1\|zinc finger protein 726 | 2 |
| gi_355390313\|ref_NP_874365.3\|protein scribble homolog isoform a | 2 |
| gi_366039979\|ref_NP_001243000.1\|E3 ubiquitin-protein ligase RNF213 isoform 3 | 1 |
| gi_37622885\|ref_NP_003860.2\|cocaine esterase isoform 1 | 1 |
| gi_381140059\|ref_NP_001244202.1\|zinc finger protein 451 isoform 3 | 1 |
| gi_38202211\|ref_NP_077288.2\|N-acetylglucosamine-1-phosphotransferase subunits alpha/beta precursor | 1 |
| gi_38788319\|ref_NP_005266.2\|guanine nucleotide-binding protein-like 1 | 1 |
| gi_38788372\|ref_NP_055506.1\|intron-binding protein aquarius | 1 |
| gi_392050772\|ref_NP_001254708.1\|zinc finger protein 850 isoform 2 | 1 |
| gi_39780552\|ref_NP_060522.3\|protein VAC14 homolog | 1 |
| gi_400153447\|ref_NP_612356.2\|zinc finger protein 551 isoform 1 | 1 |
| gi_410170757\|ref_XP_002343697.4\|PREDICTED: zinc finger protein 135 | 1 |
| gi_41281996\|ref_NP_891552.1\|HEAT repeat-containing protein 3 | 1 |
| gi_41350216\|ref_NP_061982.3\|chitobiosyldiphosphodolichol beta-mannosyltransferase | 1 |
| gi_42476320\|ref_NP_071931.2\|sushi domain-containing protein 1 precursor | 1 |
| gi_42716277\|ref_NP_055622.3\|C2 domain-containing protein 2-like | 1 |
| gi_42734325\|ref_NP_055860.1\|wings apart-like protein homolog | 1 |
| gi_4502639\|ref_NP_000570.1\|C-C chemokine receptor type 5 | 3 |
| gi_4503299\|ref_NP_003661.1\|class E basic helix-loop-helix protein 40 | 1 |
| gi_4503925\|ref_NP_002040.1\|erythroid transcription factor | 1 |
| gi_4504519\|ref_NP_001532.1\|heat shock protein beta-2 | 1 |
| gi_4505727\|ref_NP_003621.1\|peroxisomal biogenesis factor 3 | 1 |
| gi_4506243\|ref_NP_002810.1\|polypyrimidine tract-binding protein 1 isoform a | 1 |
| gi_4506259\|ref_NP_000949.1\|prostaglandin E2 receptor EP4 subtype | 1 |
| gi_4506583\|ref_NP_002936.1\|replication protein A 70 kDa DNA-binding subunit | 1 |
| gi_4507021\|ref_NP_000333.1\|band 3 anion transport protein | 1 |
| gi_4507975\|ref_NP_003410.1\|zinc finger protein 345 | 1 |
| gi_4508035\|ref_NP_003407.1\|zinc finger protein 7 | 1 |
| gi_4557587\|ref_NP_000128.1\|fumarylacetoacetase | 1 |
| gi_47157315\|ref_NP_000206.2\|tyrosine-protein kinase JAK3 | 1 |
| gi_4758874\|ref_NP_004791.1\|transmembrane 9 superfamily member 2 precursor | 1 |
| gi_48762700\|ref_NP_775808.2\|general transcription factor II-I repeat domain-containing protein 2A | 1 |
| gi_4885615\|ref_NP_005410.1\|signal transducer and activator of transcription 2 isoform 1 | 1 |
| gi_5032031\|ref_NP_005769.1\|RNA-binding protein 5 | 1 |
| gi_50428933\|ref_NP_001002259.1\|caprin-2 isoform 1 | 1 |
| gi_51317358\|ref_NP_443112.2\|phosphoinositide-3-kinase-interacting protein 1 isoform 1 precursor | 1 |
| gi_5453908\|ref_NP_006215.1\|phosphatidylinositol transfer protein alpha isoform | 1 |
| gi_54607043\|ref_NP_000148.2\|glucosylceramidase isoform 1 precursor | 1 |
| gi_55741653\|ref_NP_065982.1\|uncharacterized protein KIAA1586 | 6 |
| gi_55769564\|ref_NP_003430.1\|zinc finger protein with KRAB and SCAN domains 1 | 3 |
| gi_55769566\|ref_NP_003412.1\|zinc finger protein 37A | 2 |
| gi_56699473\|ref_NP_006289.2\|zinc finger protein with KRAB and SCAN domains 8 isoform 1 | 1 |
| gi_56788370\|ref_NP_036587.2\|PH and SEC7 domain-containing protein 4 | 2 |
| gi_57242796\|ref_NP_006609.3\|lysine-specific demethylase 5B | 1 |
| gi_5729877\|ref_NP_006588.1\|heat shock cognate 71 kDa protein isoform 1 | 1 |
| gi_57863246\|ref_NP_056084.1\|terminal uridylyltransferase 4 isoform b | 1 |
| gi_57863253\|ref_NP_067678.1\|zinc finger protein 500 | 2 |
| gi_6005942\|ref_NP_009057.1\|transitional endoplasmic reticulum ATPase | 2 |
| gi_61676195\|ref_NP_997216.2\|zinc finger protein 320 | 1 |
| gi_61743954\|ref_NP_001611.1\|neuroblast differentiation-associated protein AHNAK isoform 1 | 1 |
| gi_62739186\|ref_NP_000177.2\|complement factor H isoform a precursor | 1 |
| gi_62865871\|ref_NP_060137.2\|coiled-coil domain-containing protein 132 isoform a | 1 |
| gi_68342038\|ref_NP_001020280.1\|hematopoietic progenitor cell antigen CD34 isoform a precursor | 1 |
| gi_69885084\|ref_NP_004415.2\|transcription factor E4F1 | 1 |
| gi_71361682\|ref_NP_006176.2\|nuclear mitotic apparatus protein 1 | 1 |
| gi_71725360\|ref_NP_055857.1\|zinc finger protein 609 | 1 |
| gi_73808268\|ref_NP_071405.2\|matrix metalloproteinase-27 precursor | 1 |
| gi_74272287\|ref_NP_004985.2\|matrix metalloproteinase-9 preproprotein | 1 |
| gi_7662078\|ref_NP_055454.1\|tetratricopeptide repeat protein 37 | 1 |
| gi_7662294\|ref_NP_055620.1\|EPM2A-interacting protein 1 | 4 |
| gi_7705344\|ref_NP_057302.1\|DNA polymerase kappa | 1 |
| gi_7706683\|ref_NP_057405.1\|ammonium transporter Rh type C | 1 |
| gi_87299628\|ref_NP_683692.2\|biorientation of chromosomes in cell division protein 1-like 1 | 1 |
| gi_8922122\|ref_NP_061166.1\|zinc phosphodiesterase ELAC protein 1 | 1 |
| gi_8922430\|ref_NP_060567.1\|HAUS augmin-like complex subunit 2 isoform 1 | 1 |
| gi_94721263\|ref_NP_001035536.1\|myotubularin-related protein 12 | 1 |
| gi_9506603\|ref_NP_061946.1\|spermatogenesis-associated protein 6 precursor | 1 |
| gi_9506697\|ref_NP_061921.1\|asparagine synthetase domain-containing protein 1 | 1 |
| gi_9506931\|ref_NP_061955.1\|probable ATP-dependent RNA helicase DDX56 isoform 1 | 3 |
| gi_98961133\|ref_NP_079388.3\|zinc finger protein ZXDC isoform 1 | 1 |

Red: Genes which were not identified by the used pipeline
