# Supplementary material for: Mitochondrial Involvement in Vertebrate Speciation? The Case of Mito-nuclear Genetic Divergence in Chameleons
Source: Genome Biol Evol. 2015 Nov 19;7(12):3322–36. doi: 10.1093/gbe/evv226 (PMC4700957; doi:10.1093/gbe/evv226)
Supplement: Supplementary Data [file supp_evv226_suppl_data.zip › BarYaacov2015_Chameleons_SupplementaryTable6.docx]

| Analysis Type: | PANTHER Overrepresentation Test (release 20150430) | | | | | |
| --- | --- | --- | --- | --- | --- | --- |
| Annotation Version and Release Date: | GO Ontology database Released 2015-06-06 | | | | |  |
| Analyzed List: | Chameleon | | |  |  |  |
| Reference List: | Homo sapiens (all genes in database) | | | |  |  |
| Bonferroni correction: | TRUE |  |  |  |  |  |
| GO biological process complete | Homo sapiens - REFLIST (20814) | Chameleon (178) | Chameleon (expected) | Chameleon (over/under) | Chameleon (fold Enrichment) | Chameleon (P-value) |
| RNA metabolic process | 3,348 | 60 | 28.63 | + | 2.1 | 4.80E-05 |
| transcription, DNA-templated | 2537 | 45 | 21.7 | + | 2.07 | 1.01E-02 |
| nucleic acid-templated transcription | 2538 | 45 | 21.7 | + | 2.07 | 1.02E-02 |
| nucleic acid metabolic process | 3849 | 68 | 32.92 | + | 2.07 | 4.57E-06 |
| RNA biosynthetic process | 2657 | 46 | 22.72 | + | 2.02 | 1.45E-02 |
| gene expression | 3786 | 65 | 32.38 | + | 2.01 | 4.48E-05 |
| nucleobase-containing compound biosynthetic process | 2936 | 50 | 25.11 | + | 1.99 | 7.07E-03 |
| cellular nitrogen compound biosynthetic process | 3101 | 52 | 26.52 | + | 1.96 | 6.28E-03 |
| nucleobase-containing compound metabolic process | 4337 | 72 | 37.09 | + | 1.94 | 1.72E-05 |
| aromatic compound biosynthetic process | 3018 | 50 | 25.81 | + | 1.94 | 1.59E-02 |
| heterocycle biosynthetic process | 3018 | 50 | 25.81 | + | 1.94 | 1.59E-02 |
| regulation of cellular macromolecule biosynthetic process | 3729 | 61 | 31.89 | + | 1.91 | 1.03E-03 |
| regulation of RNA metabolic process | 3550 | 58 | 30.36 | + | 1.91 | 2.59E-03 |
| regulation of transcription, DNA-templated | 3408 | 55 | 29.14 | + | 1.89 | 8.96E-03 |
| cellular macromolecule biosynthetic process | 3540 | 57 | 30.27 | + | 1.88 | 5.59E-03 |
| cellular aromatic compound metabolic process | 4541 | 73 | 38.83 | + | 1.88 | 5.19E-05 |
| macromolecule biosynthetic process | 3614 | 58 | 30.91 | + | 1.88 | 4.74E-03 |
| regulation of nucleic acid-templated transcription | 3432 | 55 | 29.35 | + | 1.87 | 1.12E-02 |
| regulation of RNA biosynthetic process | 3452 | 55 | 29.52 | + | 1.86 | 1.34E-02 |
| regulation of nucleobase-containing compound metabolic process | 3833 | 61 | 32.78 | + | 1.86 | 2.77E-03 |
| regulation of macromolecule biosynthetic process | 3834 | 61 | 32.79 | + | 1.86 | 2.79E-03 |
| heterocycle metabolic process | 4538 | 72 | 38.81 | + | 1.86 | 1.26E-04 |
| regulation of gene expression | 4037 | 64 | 34.52 | + | 1.85 | 1.37E-03 |
| regulation of nitrogen compound metabolic process | 3931 | 62 | 33.62 | + | 1.84 | 2.89E-03 |
| cellular nitrogen compound metabolic process | 4867 | 76 | 41.62 | + | 1.83 | 7.52E-05 |
| regulation of cellular biosynthetic process | 4013 | 62 | 34.32 | + | 1.81 | 6.03E-03 |
| regulation of biosynthetic process | 4052 | 62 | 34.65 | + | 1.79 | 8.46E-03 |
| organic cyclic compound metabolic process | 4785 | 73 | 40.92 | + | 1.78 | 5.07E-04 |
| nitrogen compound metabolic process | 5241 | 78 | 44.82 | + | 1.74 | 3.74E-04 |
| cellular biosynthetic process | 4448 | 64 | 38.04 | + | 1.68 | 4.42E-02 |
| regulation of macromolecule metabolic process | 5396 | 75 | 46.15 | + | 1.63 | 1.45E-02 |
| regulation of primary metabolic process | 5414 | 74 | 46.3 | + | 1.6 | 3.48E-02 |
| regulation of cellular metabolic process | 5661 | 76 | 48.41 | + | 1.57 | 4.67E-02 |
| cellular macromolecule metabolic process | 6534 | 87 | 55.88 | + | 1.56 | 6.70E-03 |
| macromolecule metabolic process | 7230 | 94 | 61.83 | + | 1.52 | 4.51E-03 |
| primary metabolic process | 8526 | 107 | 72.91 | + | 1.47 | 1.55E-03 |
| organic substance metabolic process | 8850 | 107 | 75.68 | + | 1.41 | 1.33E-02 |
| metabolic process | 9942 | 118 | 85.02 | + | 1.39 | 3.63E-03 |
| Unclassified | 4304 | 19 | 36.81 | - | 0.52 | 0.00E+00 |
