# Supplementary material for: Mitochondrial Involvement in Vertebrate Speciation? The Case of Mito-nuclear Genetic Divergence in Chameleons
Source: Genome Biol Evol. 2015 Nov 19;7(12):3322–36. doi: 10.1093/gbe/evv226 (PMC4700957; doi:10.1093/gbe/evv226)
Supplement: Supplementary Data [file supp_evv226_suppl_data.zip › BarYaacov2015_Chameleons_SupplementaryTable7.docx]

| Analysis Type: | PANTHER Overrepresentation Test (release 20150430) | | | | | |
| --- | --- | --- | --- | --- | --- | --- |
| Annotation Version and Release Date: | GO Ontology database Released 2015-06-06 | | | | |  |
| Analyzed List: | Chameleon | | |  |  |  |
| Reference List: | Homo sapiens (all genes in database) | | | |  |  |
| Bonferroni correction: | TRUE |  |  |  |  |  |
| GO molecular function complete | Homo sapiens - REFLIST (20814) | Chameleon (178) | Chameleon (expected) | Chameleon (over/under) | Chameleon (fold Enrichment) | Chameleon (P-value) |
| nucleic acid binding transcription factor activity | 1119 | 29 | 9.57 | + | 3.03 | 2.51E-04 |
| sequence-specific DNA binding transcription factor activity | 1119 | 29 | 9.57 | + | 3.03 | 2.51E-04 |
| DNA binding | 2419 | 49 | 20.69 | + | 2.37 | 1.41E-05 |
| nucleic acid binding | 3928 | 74 | 33.59 | + | 2.2 | 5.97E-09 |
| metal ion binding | 4071 | 67 | 34.81 | + | 1.92 | 4.10E-05 |
| cation binding | 4147 | 67 | 35.46 | + | 1.89 | 8.66E-05 |
| heterocyclic compound binding | 5811 | 89 | 49.7 | + | 1.79 | 9.52E-07 |
| organic cyclic compound binding | 5884 | 89 | 50.32 | + | 1.77 | 1.89E-06 |
| ion binding | 6052 | 88 | 51.76 | + | 1.7 | 2.13E-05 |
| binding | 13913 | 153 | 118.98 | + | 1.29 | 1.26E-05 |
| molecular_function | 16624 | 165 | 142.17 | + | 1.16 | 4.43E-03 |
| Unclassified | 4190 | 13 | 35.83 | - | 0.36 | 0.00E+00 |
