# Supplementary material for: Mitochondrial Involvement in Vertebrate Speciation? The Case of Mito-nuclear Genetic Divergence in Chameleons
Source: Genome Biol Evol. 2015 Nov 19;7(12):3322–36. doi: 10.1093/gbe/evv226 (PMC4700957; doi:10.1093/gbe/evv226)
Supplement: Supplementary Data [file supp_evv226_suppl_data.zip › BarYaacov2015_Chameleons_SupplementaryTable8.docx]

| Analysis Type: | PANTHER Overrepresentation Test (release 20150430) | | | | | |
| --- | --- | --- | --- | --- | --- | --- |
| Annotation Version and Release Date: | GO Ontology database Released 2015-06-06 | | | | |  |
| Analyzed List: | Chameleon | | |  |  |  |
| Reference List: | Homo sapiens (all genes in database) | | | |  |  |
| Bonferroni correction: | TRUE |  |  |  |  |  |
| GO cellular component complete | Homo sapiens - REFLIST (20814) | Chameleon (178) | Chameleon (expected) | Chameleon (over/under) | Chameleon (fold Enrichment) | Chameleon (P-value) |
| nucleus | 6603 | 97 | 56.47 | + | 1.72 | 3.34E-07 |
| organelle lumen | 4012 | 57 | 34.31 | + | 1.66 | 4.17E-02 |
| intracellular membrane-bounded organelle | 10413 | 126 | 89.05 | + | 1.41 | 1.70E-05 |
| membrane-bounded organelle | 11586 | 138 | 99.08 | + | 1.39 | 1.13E-06 |
| intracellular organelle | 11434 | 134 | 97.78 | + | 1.37 | 1.79E-05 |
| organelle | 12479 | 144 | 106.72 | + | 1.35 | 2.07E-06 |
| intracellular part | 13190 | 145 | 112.8 | + | 1.29 | 1.25E-04 |
| intracellular | 13534 | 147 | 115.74 | + | 1.27 | 1.94E-04 |
| Unclassified | 3159 | 10 | 27.02 | - | 0.37 | 0.00E+00 |
