# Supplementary material for: Mitochondrial Involvement in Vertebrate Speciation? The Case of Mito-nuclear Genetic Divergence in Chameleons
Source: Genome Biol Evol. 2015 Nov 19;7(12):3322–36. doi: 10.1093/gbe/evv226 (PMC4700957; doi:10.1093/gbe/evv226)
Supplement: Supplementary Data [file supp_evv226_suppl_data.zip › BarYaacov2015_Chameleons_SupplementaryTable9.docx]

| **Number** | **Orientation** | **Gene** | **Sequence** |
| --- | --- | --- | --- |
| 1 | F | POLRMT | GCCCGTTTCATTGCCAAATCTG |
| 2 | R | POLRMT | GTGGTATCGTGGCTCCATTTG |
| 3 | R | POLRMT | GAGAAGATGCTAAAAGGCTGAC |
| 4 | F | POLRMT | GATGGAACTGTTGTCTAGCATC |
| 5 | R | POLRMT | GCTGAAAAAATATGTTGATT |
| 6 | F | POLRMT | AGAAAGATTGCTGTATAAAGCC |
| 7 | R | POLRMT | CAGCGGCAGAAAGACAGAGATG |
| 8 | F | SDHC | CCAATGGGAACAACAGCAAAAG |
| 9 | R | SDHC | CATTGCAATTCCTGTACCCCG |
| 10 | R | SDHC | GGATTGAAAGGATTTTTGCTGG |
| 11 | F | MARS | CCAGAGGATGCATCACAAACC |
| 12 | R | MARS | CACCGGTTGTAGCAGGATCCC |
| 13 | F | NDUFA5 | TGTGGACAAATAGAGGAAGTG |
| 14 | R | NDUFA5 | ATTGGCCATTTCCATTGGTC |
| 15 | F | NDUFA5 | CAGCATATTGGGGGCAAAATG |
| 16 | F | MRPL30 | TGCGGTAGGGGGCGATTGCTC |
| 17 | R | MRPL30 | ATTCAGGAACTCTTGCTTTTG |
| 18 | R | MRPL30 | CGACTCTGGGCGGCTTACAAC |
| 19 | F | ACAD9 | TTTGCATGATACCTTTGGTCG |
| 20 | R | ACAD9 | GCCAAACCTCCTTAGCAAGCC |
| 21 | F | ACAD9 | GAAACCACGGAAAACAGCAAAC |
| 22 | F | ACAD9 | AATAAGGCTGAATCCATAACG |
| 23 | F | TCIRG1 | CTCTCAGAGGTGCTTTGGAG |
| 24 | R | TCIRG1 | CGTAAAGCATGCAAAAAAGC |
| 25 | F | AARS2 | TTGACGGGAGAGCAAGCCAAG |
| 26 | R | AARS2 | GCCAATCTCCTTAGCCAGTC |
| 27 | F | AARS2 | GTCTTAGGCCCGAGAAGCTGG |
| 28 | F | P32 | GAATTCCTGACAGATGAAATC |
| 29 | R | P32 | GCTACTTTCCGGGTAAGCTTG |
| 30 | F | ACO1 | ACAAACCCACAAGGAAAGAAG |
| 31 | R | ACO1 | TTTTGGTAGACTTCTTTAAAC |
| 32 | F | ETFA | CTCAACATGATGCCTACAAAG |
| 33 | R | ETFA | GCAGATGCTCCGACACAGATG |
| 34 | F | ETFA | CAGCTTCGCATTAAAGTTTCC |
| 35 | F | LYRM4 | GCCATAAGAAGAATAAGAGATGCC |
| 36 | R | LYRM4 | CTAGATTAGTTCTTGCTTTGC |
| 37 | F | LYRM4 | CTGTTGCGAGAGAGCCAGGGC |
| 38 | R | LYRM4 | TAGAATAGAGTTGGCCAATGG |
