# Supplementary figures and images for: Mitochondrial Involvement in Vertebrate Speciation? The Case of Mito-nuclear Genetic Divergence in Chameleons
Source: Genome Biol Evol. 2015 Nov 19;7(12):3322–36. doi: 10.1093/gbe/evv226 (PMC4700957; doi:10.1093/gbe/evv226)

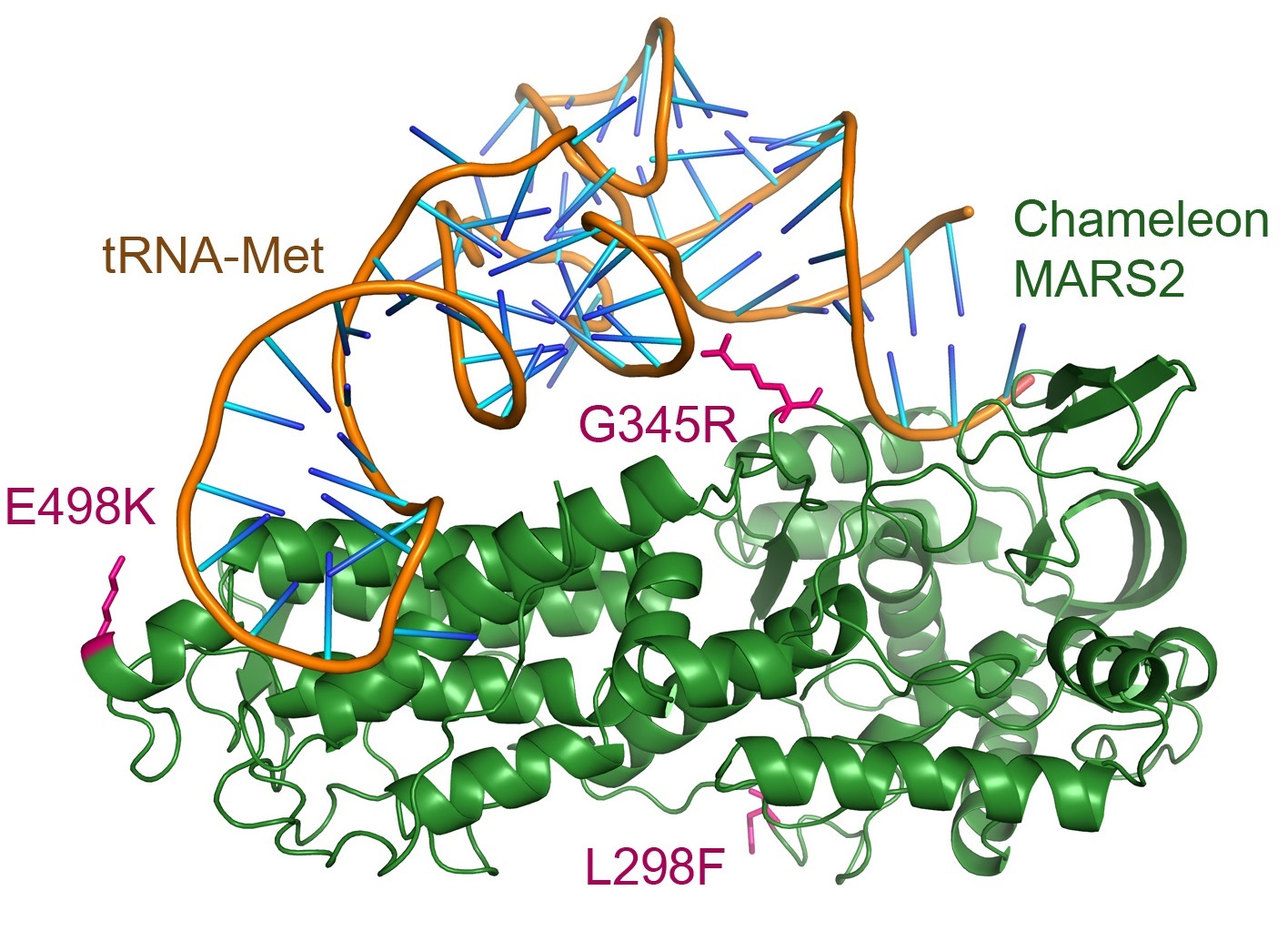

Supplement: Supplementary Data [file supp_evv226_suppl_data.zip › BarYaacov2015_Chameleons_Supplemnetary Figure 1_MARS2.jpg]

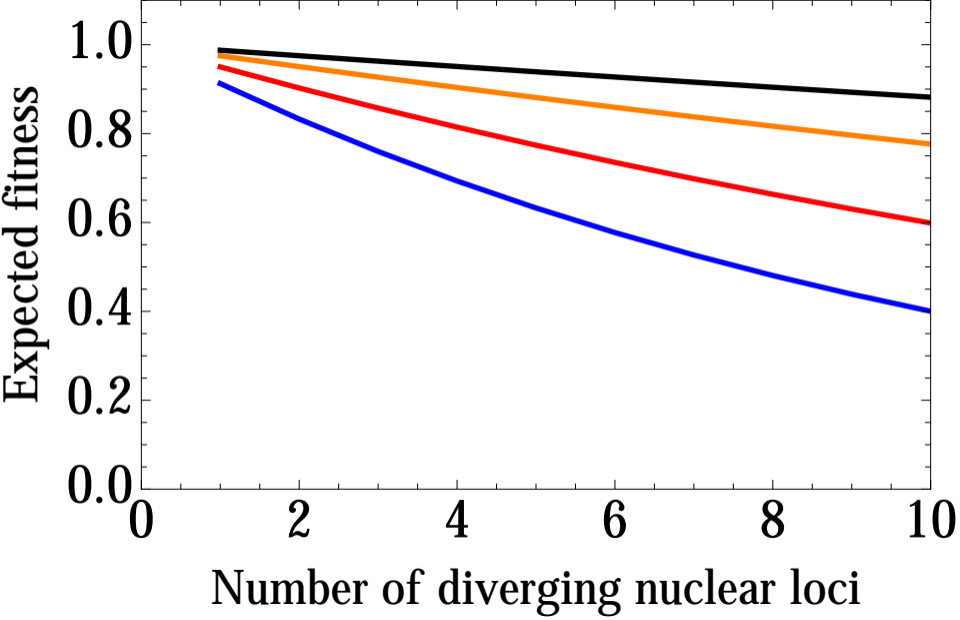

Supplement: Supplementary Data [file supp_evv226_suppl_data.zip › BarYaacov2015_Chameleons_Supplemnetary Figure 2.pdf]
